# Sema7A is crucial for resolution of severe inflammation

**DOI:** 10.1073/pnas.2017527118

**Published:** 2021-02-26

**Authors:** Andreas Körner, Alice Bernard, Julia C. Fitzgerald, Juan Carlos Alarcon-Barrera, Sarantos Kostidis, Torsten Kaussen, Martin Giera, Valbona Mirakaj

**Affiliations:** ^a^Department of Anesthesiology and Intensive Care Medicine, Molecular Intensive Care Medicine, University Hospital Eberhard-Karls University, 72076 Tübingen, Germany;; ^b^Hertie Institute for Clinical Brain Research, University Clinic Tübingen, 72076 Tübingen, Germany;; ^c^Center for Proteomics and Metabolomics, Leiden University Medical Center, 2333 ZA, Leiden, The Netherlands;; ^d^Department of Pediatric Cardiology and Pediatric Intensive Care Medicine, Hannover Medical School, 30625 Hannover, Germany

**Keywords:** Semaphorin 7A, inflammation, metabolism, lipid mediator, resolution

## Abstract

Endogenous mediators regulating acute inflammatory responses in both the induction and resolution phases of inflammatory processes are pivotal in host defense and tissue homeostasis. Recent studies have identified neuronal guidance proteins characterized in axonal development that display immunomodulatory functions. Here, we identify the neuroimmune guidance cue Semaphorin 7A (Sema7A), which appears to link macrophage (MΦ) metabolic remodeling to inflammation resolution. Sema7A orchestrated MΦ chemotaxis and chemokinesis, activated MΦ differentiation and polarization toward the proresolving M2 phenotype, and promoted leukocyte clearance. Peritoneal MΦ^Sema7A−/−^ displayed metabolic reprogramming, characterized by reductions in fatty acid oxidation and oxidative phosphorylation, increases in glycolysis and the pentose phosphate pathway, and truncation of the tricarboxylic acid cycle, which resulted in increased levels of the intermediates succinate and fumarate. The low accumulation of citrate in MΦ^Sema7A−/−^ correlated with the decreased synthesis of prostaglandins, leading to a reduced impact on lipid-mediator class switching and the generation of specialized pro resolving lipid mediators. Signaling network analysis indicated that Sema7A induced the metabolic reprogramming of MΦ by activating the mTOR- and AKT2-signaling pathways. Administration of Sema7A^SL4cd^ orchestrated the resolution response to tissue homeostasis by shortening the resolution interval, promoting tissue protection in murine peritonitis, and enhancing survival in polymicrobial sepsis.

## Significance

Nonresolving inflammation, a hallmark of sepsis and/or multi-organ failure, still poses a challenge in medicine. The mortality rate is enormous, and so far no adequate curative therapy is available. Here we identify a previously unrecognized role of the neuronal guidance protein semaphorin 7A in the transition to resolution processes in severe systematic inflammation such as sepsis.

Acute inflammatory responses underlie fundamental pathophysiological mechanisms to protect the host; however, when these processes become out of control, acute inflammation can lead to collateral tissue destruction and loss of functional organ integrity. The control of excessive leukocyte recruitment is critical during the initial immune response, and in the context of inflammation resolution, adequate clearance is essential for the final restoration of tissue homeostasis. However, recent evidence indicates that immune cells and metabolic systems are highly coordinated with each other and that this process is critically important for defining cellular function and fate (1, 2). Cells such as monocytes and macrophages (MΦ), which are central modulators/adjustors in the maintenance of tissue homeostasis and repair, undergo metabolic reprogramming in response to inflammatory processes to meet cellular demands such as phagocytosis, proliferation, and cytokine release.

A series of discoveries have revealed biological parallels between the nervous and immune systems in which neuronal guidance proteins (NGP) function during important immunological processes (3). Semaphorin 7A (Sema7A), one such guidance cue, is a glycosylphosphatidylinositol (GPI)-anchored membrane protein with chemoattractant and chemorepulsive attributes. In addition to its role in guiding axon pathfinding during neuronal development, Sema7A has diverse functions in morphogenesis and immune cell control (3) and regulates T cell responses via the α1β1 integrin receptor (4). During hypoxia, endothelial Sema7A was observed to induce extravascular neutrophil migration by interacting with the plexin C1 receptor, thus exerting proinflammatory effects (5). Protective and antiinflammatory effects were observed by the interaction with integrin receptors in dextran sulfate sodium-induced colitis (6). It appears that Sema7A may exert opposite effects by interacting with its different receptors and organ systems. During an acute inflammatory response against invading pathogens, complete resolution of tissue inflammation is the ideal outcome for tissue recovery and functional integrity. The resolution of inflammation is an active process and is considered to be separate from antiinflammatory processes (7, 8). Considering the numerous findings showing the impact of Sema7A, particularly in the initial phase of inflammation, we here aimed to investigate the role of Sema7A in the metabolic reprogramming of MΦ and the transition to resolution processes during severe inflammation. Here, we demonstrate that metabolic reprogramming in peritoneal MΦ^Sema7A−/−^, which showed an increased energy demand compared to that of MΦ^Sema7A+/+^, reflected by an increased glucose intake, increased glycolysis rate and up-regulated pentose phosphate pathway (PPP). Protein microarray analysis revealed suppression of mTOR signaling and activation of phosphorylated AKT2 signaling in peritoneal MΦ^Sema7A−/−^. Both are critical for the inflammatory response, as they lower oxidative phosphorylation (OXPHOS) levels, activate NF-κB–mediated transcription, and trigger M1 polarization (9).

The reduced accumulation of the tricarboxylic acid cycle (TCA) intermediate citrate in MΦ^Sema7A−/−^ correlated with decreased synthesis of the prostaglandins PGD_2_ and PGE_2_ in Sema7A^−/−^ peritoneal exudates, leading to a reduced impact on lipid-mediator class switching and, ultimately, to reduced generation of specialized proresolving lipid mediators (SPMs), such as LXA_4_ and PDX, and their pathway metabolites. Studies of murine peritonitis showed that Sema7A reduced severe inflammatory peritonitis, shortened the resolution interval, stimulated the generation of SPMs, promoted MΦ clearance, stimulated tissue regeneration, decreased mortality rates, and enhanced the survival of mice with polymicrobial sepsis.

Since the arginine-glycine-aspartic acid (RGD) motif seemed to be mandatory for the interaction of Sema7A with its specific receptors PlexinC1 and the integrins in particular, in experiments we used the Sema7A SL4cd peptide, a peptide comprising 19 amino acids of the SL4cd region from murine Sema7A.

The results here reveal crucial roles of Sema7A in linking metabolic reprogramming and the resolution of inflammation, potentially furthering our understanding of the inflammation resolution processes in innervated organs as well as providing therapeutic value for the treatment of acute inflammatory conditions.

## Results

### Sema7A Controls the Macrophage Inflammatory Functional Phenotype by Shifting Cells from the M1 to the M2 Phenotype.

**Fig. 1.**
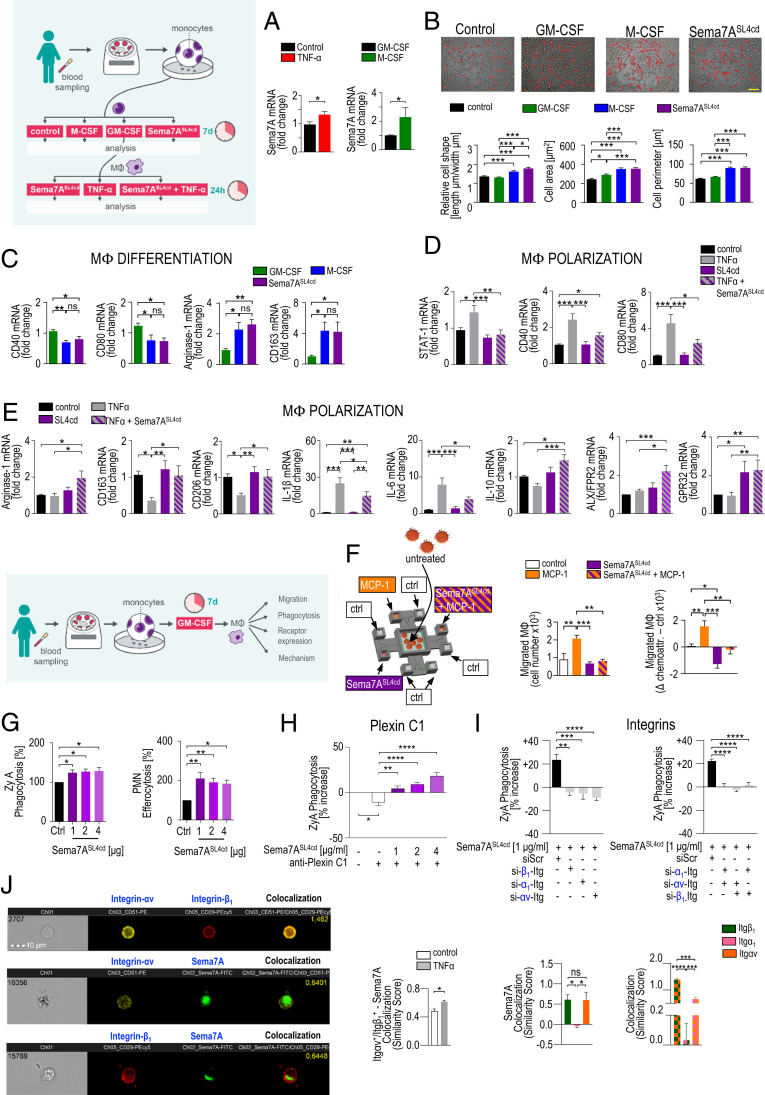
Sema7A controls the macrophage inflammatory phenotype and regulates human macrophage chemotaxis and chemokinesis. Human PBMCs were stimulated with GM-CSF, M-CSF, or Sema7A^SL4cd^ for 7 d, and Sema7A transcript expression in differentiated M1 and M2 MΦs was quantified by RT-PCR (*n* = 14 to 16) (*A*). (*B*) Cell morphology was analyzed by phase contrast images and measurements of the cell shape, length, and perimeter (magnification 200×). (Scale bar: 20 μm.) *n* = 210. (*C*) The expression levels of key genes that contribute to M2 differentiation, Arg1 and CD163, and central genes of M1 differentiation, STAT-1 and CD80, were analyzed (*n* = 11). (*D* and *E*) Phenotypic polarization of macrophages: M1 MΦs were sequentially challenged with Sema7A^SL4cd^ and TNF-α or vehicle for 24 h. The gene expression levels of M1 polarization markers (including STAT-1, CD40, CD80, IL-1β, and IL-6), key genes of M2 polarization (such as Arg1, CD163, CD206, and IL-10) and the ALX/FPR2 and GPR32 receptors were quantified by RT-PCR (*n* = 13 to 20). (*F*) Schematic model of the microfluidic migration chamber. A chemoattractive gradient with a monocyte chemotactic protein (MCP-1) and Sema7A^SL4cd^ was established between eight peripheral wells and a central cell loading well. M1 MΦ chemotaxis was evaluated using a Casy TT cell counter (Omni Life Science) (*n* = 10). (*G*) The rate of MΦ clearance of fluorescence-labeled ZyA particles and human apoptotic PMNs was assessed photometrically (*n* = 5 to 9). (*H* and *I*) MΦs were transfected with integrin α_1_-, integrin αv-, or integrin β_1_-siRNA or treated with an anti-Plexin C1 and then stimulated with Sema7A^SL4cd^ antibody. MΦ clearance of fluorescence-labeled ZyA particles was then assessed photometrically (*n* = 4 to 10). (*J*) The colocalization of integrin heterodimers and integrins with Sema7A was assessed with the Similarity feature of the ImageStreamx mkII system. The results are representative of three to eight independent experiments and are expressed as the mean ± SEM; significance was determined by one-way ANOVA with Bonferroni correction (*B*–*I*) or the unpaired two-tailed Student’s *t* test (*A*). *P < 0.05, **P < 0.01, ***P < 0.001, ****P < 0.0001.

Evidence suggests that the monocyte and MΦ lineages are of central importance for the resolution of inflammation (10–12). In response to environmental signals derived from inflammatory tissues or activated cells or microbes, these cells differentiate or polarize into the classical proinflammatory M1, alternative antiinflammatory M2, or intermediate M2 phenotype (11). We show that the Sema7A messenger RNA (mRNA) levels were higher in M-CSF–stimulated M2 MΦs than in GM-CSF–stimulated M1 MΦs (Fig. 1*A*). Next, we analyzed the cell shapes that were recently described as indicators of differentiation in M1 and M2 phenotypes (13). As expected, GM-CSF stimulated M1 MΦs with a specific round shape, M-CSF stimulated M2 MΦs with a particular elongated cell shape, and, interestingly, MΦs stimulated with Sema7A^SL4cd^ (*SI Appendix*, Fig. S1 *A*–*C*) showed an elongated M2 morphology (Fig. 1*B* and *SI Appendix*, Fig. S1*D*). In a different experimental setup, M1 differentiation was induced by stimulation with GM-CSF for 7 d and then with LPS and IFNγ (±Sema7A^SL4cd^) for 2 d. M2 differentiation was initiated by stimulating MΦs with M-CSF for 7 d and then with IL-4 and Il-13 (±Sema7A^SL4cd^) for 2 d. These findings were substantiated by key genes and proteins contributing to M1/M2 differentiation (Fig. 1*C* and *SI Appendix*, Fig. S1*E*). To investigate the direct influence of Sema7A on the phenotypic polarization of MΦs, MΦs were exposed to Sema7A^SL4cd^ and subsequently stimulated with TNF-α or vehicle for 24 h. Our data revealed a significant reduction in M1 markers (STAT-1, CD40, CD80) and inflammatory cytokines, whereas M2 markers (Arg1, CD163, and CD206) and IL-10 were strongly increased by Sema7A^SL4cd^ at the RNA and protein levels (Fig. 1 *D* and *E* and *SI Appendix*, Fig. S1*F*). This was associated with increased MΦ expression of the G-protein–coupled receptors ALX/FPR2 and GPR32, which are known to mediate proresolving actions (14) (Fig. 1*E*), implying that Sema7A shifted the polarization state toward the M2 phenotype and thus presumably promoted resolution and metabolic homeostasis (12).

### Sema7A Reduces M1 MΦ Chemotaxis and Chemokinesis and Enhances Macrophage Phagocytosis by Interacting with Integrin Receptors.

Next, we sought to investigate the effect of Sema7A on the control of MΦ chemotaxis and chemokinesis. Using a microfluidic chamber as described in *SI Appendix*, Fig. S2*A*, Sema7A^SL4cd^ was shown to strongly decrease MΦ chemotaxis to MCP-1, but in the absence of the chemoattractant MCP-1, Sema7A^SL4cd^ had an independent repulsive effect on M1 MΦ migration, suggesting that Sema7A strongly influenced M1 MΦ recruitment by reducing both chemotaxis and chemokinesis (Fig. 1*F*). One key attribute of inflammation resolution is the clearance of apoptotic cells, microorganisms, cell debris, and particles at the inflamed site, and our in vitro phagocytosis and efferocytosis studies reveal that Sema7A significantly enhances the clearance of zymosan A (ZyA) particles and apoptotic polymorphonuclear cell (PMNs) (Fig. 1*G*). To better understand the mechanism by which Sema7A mediates these proresolving phagocytic effects, we focused on the most specific receptors, plexin C1 and integrin (Itg) (3). First, we investigated the expression of the plexin C1 receptor and integrin heterodimers in diverse organs, such as the liver, lung, and peritoneum, and observed differential expression patterns of the receptors in peritoneal MΦs, Kupffer cells, and alveolar MΦs, which might explain the pro- and antiinflammatory effects of Sema7A in different organs (*SI Appendix*, Fig. S2*B*). We performed phagocytosis experiments in which human MΦs were incubated with Sema7A^SL4cd^ and/or an anti–Plexin-C1 antibody and found that the phagocytic impact of Sema7A was not affected by Plexin C1 (Fig. 1*H*). Next, we focused on the integrin receptor and its heterodimers. For this, we transfected human MΦs with small interfering RNA (siRNA) targeting α_1_Itg, αvItg, or β_1_Itg and subsequently stimulated them with Sema7A^SL4cd^ (Fig. 1*I*). The results showed that the phagocytic effect of Sema7A was significantly reduced in MΦ cells transfected with the integrin heterodimers (Fig. 1*I*). To substantiate these results, we performed image stream analyses, which showed colocalization of the heterodimers αv integrin and β_1_ integrin with Sema7A, revealing the strongest binding on the monocyte/MΦ surface (Fig. 1*J* and *SI Appendix*, Fig. S2 *C*–*E*)

### Sema7A Regulates MΦ Immunometabolism.

**Fig. 2.**
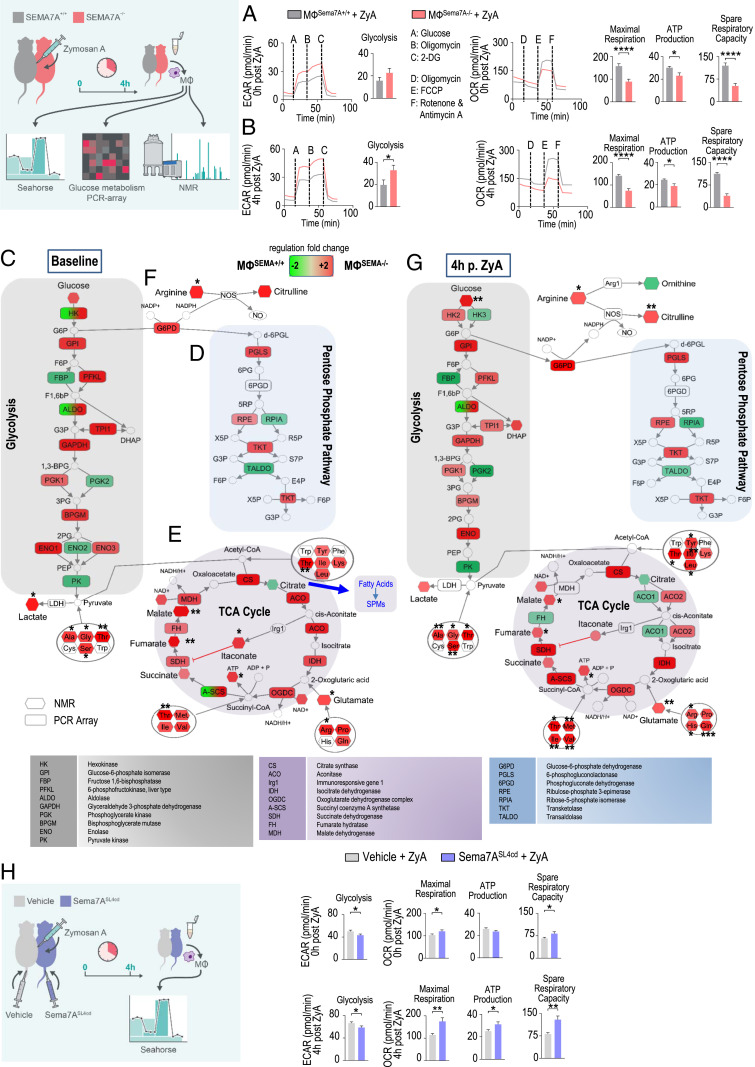
Sema7A drives cellular metabolism. Glycolytic capacity and mitochondrial respiration were assessed by using the Seahorse Glycolysis and Mito Stress tests. MФs^SEMA7A+/+^ and MФs^SEMA7A−/−^ were subjected to peritoneal lavage, and ECARs were measured after consecutive injections of glucose, oligomycin, and 2-DG. Oxygen consumption rates were determined after injections of oligomycin, FCCP, rotenone, and antimycin A. Glycolytic and mitochondrial respiration markers were calculated (*A*) at baseline and (*B*) following stimulation with ZyA for 4 h (*n* = 20). The glucose metabolism pathways in MФs^SEMA7A+/+^ and MФs^SEMA7A−/−^ were assessed via the PCR-based analysis of enzymes involved in glucose metabolism. Intracellular metabolites were quantified by NMR spectroscopy (*n* = 3). The experiment was conducted (*C*–*F*) at baseline and (*G*) following stimulation with ZyA for 4 h. (*H*) ECAR and OCR were also determined in MФs from C57/BL/6 mice that were stimulated either with vehicle (MФ^vehicle^) or the peptide Sema7A^SL4cd^ (MФ^SEMA7A−SL4cd^) followed by ZyA stimulation for 0 and 4 h. Samples were pooled from three to four mice in each group. The results represent three independent experiments and are expressed as the mean ± SEM; significance was determined by the unpaired two-tailed Student’s *t* test. *P < 0.05; **P < 0.01; ***P < 0.001, ****P < 0.0001.

**Fig. 3.**
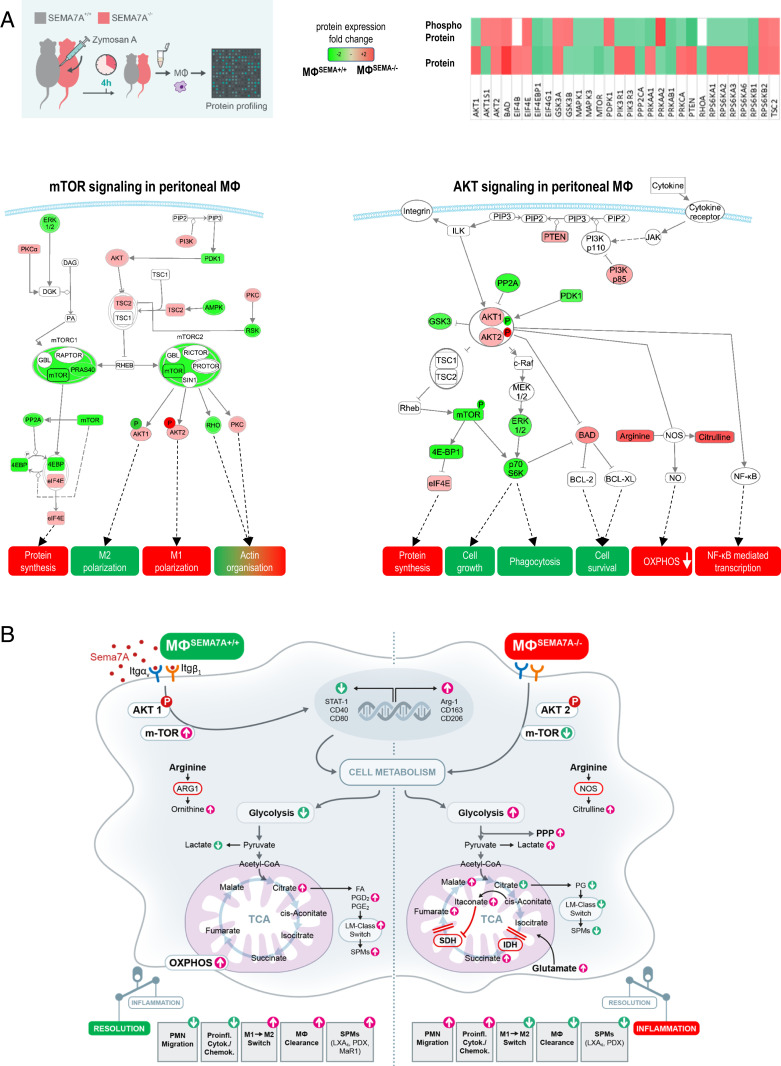
Sema7A-dependent macrophage intracellular signaling. SEMA7A^+/+^ and SEMA7A^−/−^ animals were injected intraperitoneally with ZyA, and peritoneal MΦs were collected 4 h later. (*A*) The mTOR- and AKT-signaling pathways were assessed by protein expression and phosphorylation analysis by using a protein microarray. Samples were pooled from three to four mice in each group for each experiment. (*B*) Schematic model of the cellular effects of Sema7A.

**Fig. 4.**
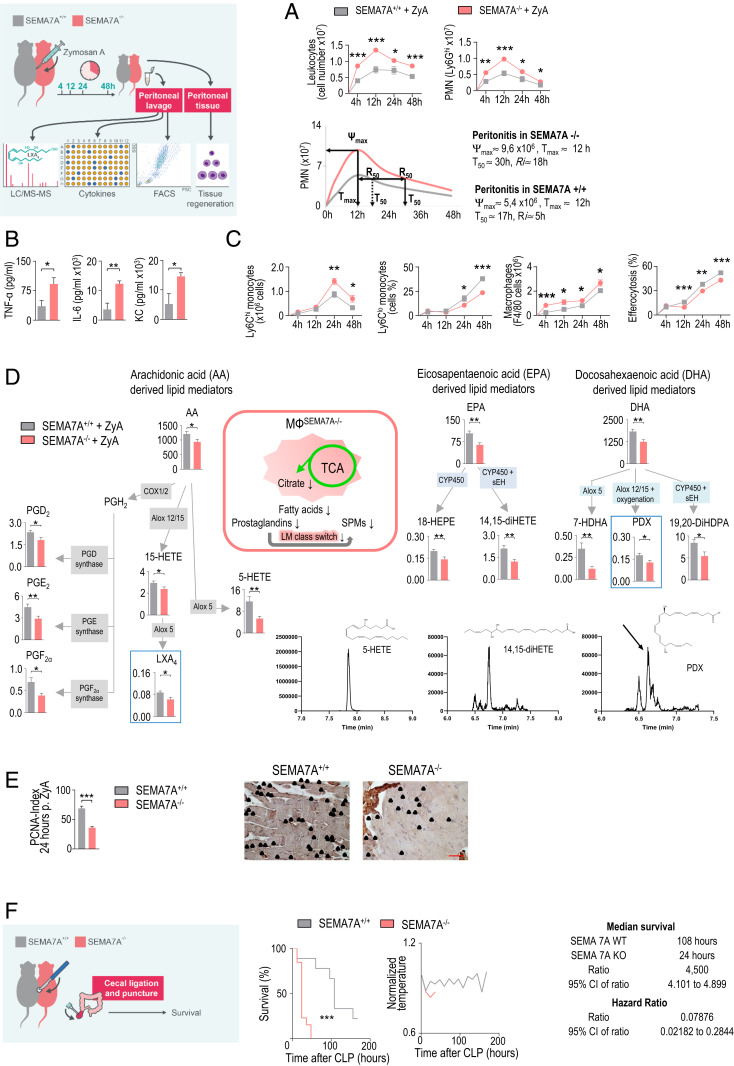
SEMA7A^−/−^ mice display deficient inflammation resolution. Sema7A-deficient mice and littermate controls were injected with ZyA, and peritoneal lavages were collected at 4, 12, 24, and 48 h. (*A*) Total leukocytes were counted by light microscopy, and PMNs were identified by flow cytometry (*n* = 6 to 16). Resolution indices. (*B*) The cytokine levels of IL-1β, IL-6, and KC in peritoneal fluids were measured by enzyme-linked immunosorbent assay (ELISA) (*n* = 5 to 6). (*C*) Classical and nonclassical monocytes and MΦs as well as monocyte-derived MΦ efferocytosis were quantified by flow cytometry (*n* = 6 to 16). (*D*) The levels of bioactive lipid mediators and precursors derived from arachidonic acid (AA), docosahexaenoic acid (DHA), and eicosapentaenoic acid (EPA) in the peritoneal fluids of SEMA7A^+/+^ and SEMA7A^−/−^ animals treated with ZyA (*n* = 15 to 19) for 4 h were quantified by LC-MS/MS–based profiling. (*E*) PCNA expression in peritoneal slices (24 h after ZyA injection) as detected by immunohistochemistry (*n* = 4) and the calculated indices (40× magnification). (Scale bar: 50 µm.) (*F*) Survival rates and normalized body temperatures of SEMA7A^+/+^ and SEMA7A^−/−^ animals that underwent the CLP procedure with median survival and hazard ratios (WT: *n* = 9; KO: *n* =13). The results represent at least two independent experiments and are expressed as the mean ± SEM (*A*–*E*) and the geometric mean (*F*); significance was determined by the unpaired two-tailed Student’s *t* test (*A*–*E*) or log-rank test (*F*). *P < 0.05; **P < 0.01; ***P < 0.001.

To investigate whether the changes in MΦ phenotype profiles were associated with alterations in cellular energy metabolism, we collected murine residential peritoneal MΦs^Sema7A−/−^ and determined the extracellular acidification rate (ECAR), a measurement of lactic acid production, by Seahorse extracellular flux analysis. As shown in Fig. 2*A* and *SI Appendix*, Fig. S3*A*, increased basal ECARs and reduced mitochondrial oxygen consumption rates (OCRs) were observed in peritoneal MΦ^Sema7A−/−^, as indicated by decreases in the maximal respiration, mitochondrial ATP production, and spare respiratory capacity compared with those in the wild-type (WT) controls. In a second experimental setting, we pretreated peritoneal MΦs^Sema7A−/−^ with ZyA for 4 h and observed identical expression (Fig. 2*B*), suggesting that the deficiency of Sema7A shifted MΦ metabolism toward aerobic glycolysis and reduced OXPHOS (Fig. 2*B* and *SI Appendix*, Fig. S3*B*). To further understand the metabolism programs affected by Sema7A, we performed an NMR-based quantitative metabolomics analysis and a glucose metabolism PCR array of peritoneal MΦs^Sema7A−/−^ and the corresponding controls. In MΦs^Sema7A−/−^, we found enhanced glycolysis at the baseline as determined by the increased expression of genes encoding key regulators, such as hexokinase 2 (HK2), glyceraldehyde 3-phosphate dehydrogenase (GAPDH), 6-phosphofructokinase (PFKL), and enolase 1 (ENO1; expressed on the cell surface of inflammatory MΦs) (*SI Appendix*, Table S1), ultimately leading to increased lactate production (Fig. 2*C*) (1, 15). Enhanced glycolysis was linked to induction of the PPP (Fig. 2*D*), as evidenced by the expression of glucose-6-phosphate dehydrogenase (G6PD), 6-phosphogluconolactonase (PGLS), ribulose-phosphate-3-epimerase (RPE), and transketolase (TKT) (16). Next, we focused on the TCA cycle (Fig. 2*E*) and found it to be interrupted at the isocitrate dehydrogenase (IDH) and succinate dehydrogenase (SDH) steps, leading to the accumulation of succinate and other TCA intermediates such as fumarate and malate in MΦs^Sema7A−/−^. In particular, citrate synthase and IDH were strongly induced, which did not lead to citrate accumulation but was possibly due to the provision of citrate consumption for the synthesis of itaconate and nitric oxide (NO). In fact, we found an accumulation of itaconate, which is thought to specifically initiate succinate accumulation in M1 MΦs, as it inhibits SDH and may therefore play a serious role in the switch to glycolysis (Fig. 2*E* and *SI Appendix*, Table S2) (1, 17). The decreased levels of citrate correlated with our lipid mediator profile where PGD_2_ and PGE_2_ were strongly decreased in Sema7A^−/−^ peritoneal exudates, indicating a reduced impact on lipid-mediator class switching (Fig. 3*B*) and, ultimately, on the generation or SPMs such as LXA_4_ and PDX (Fig. 4*D*). Another metabolic signature inducing succinate and fumarate accumulation in MΦs^Sema7A−/−^ was derived from glutamine metabolism. Of note, glutamine metabolism has been shown to play a central role in the induction of trained immunity, where it displays increased levels in “trained” monocytes/MΦs and leads to succinate and fumarate accumulation (18). An additional factor indicating that MΦs^Sema7A−/−^ displayed the M1 phenotype was the impact on arginine metabolism, where arginine was activated to NO and citrulline (Fig. 2*F*). In the next experimental setting, MΦs^Sema7A−/−^ were pretreated with ZyA for 4 h, followed by NMR-based metabolomic profiling and a glucose metabolism PCR array. The data revealed very similar metabolic alterations in MΦs^Sema7A−/−^ treated with and without ZyA (Fig. 2*G* and *SI Appendix*, Tables S1 and S2), and this pattern was reversed upon treatment with Sema7A^SL4cd^. Administration of Sema7A^SL4cd^ (±ZyA) improved mitochondrial function in peritoneal MΦs, as evidenced by significant increases in the maximal respiration, mitochondrial MΦ ATP production, and spare respiratory capacity (Fig. 2*H* and *SI Appendix*, Fig. S3 *C* and *D*).

### Sema7A-Dependent MΦ Intracellular Signaling.

Having demonstrated the phagocytic impact of Sema7A induction via interaction with the α_v_β_1_ integrin, we next aimed to investigate downstream signaling. Murine peritoneal MΦs^Sema7A−/−^ and MΦs^Sema7A+/+^ were collected 4 h after the induction of peritonitis for analysis. We performed a protein microarray to determine the levels of expression and, accordingly, phosphorylation of these proteins. The data showed that Sema7A activated the mTOR and AKT1 phosphorylation-signaling pathways, which are known to be important polarizing signals in regulating MΦ metabolism and polarization toward the M2 phenotype (19) (Fig. 3 *A* and *B* and *SI Appendix*, Fig. S4 *A* and *B* and Dataset S1). Furthermore, mTORC2 activation plays a crucial role in the proliferation, differentiation, and survival of M2 MΦs (15).

### Sema7A-Deficient Mice Display Impaired Resolution Features and Worse Survival.

Next, we exposed Sema7A^−/−^ mice and their littermate controls to ZyA-induced peritonitis. At 4, 12, 24, and 48 h, we collected peritoneal lavages to monitor the onset and resolution phases of acute inflammation. Sema7A^+/+^ mice exhibited maximal PMN infiltration at 12 h, followed by a decrease, yielding a resolution interval (R*i*) of 5 h, whereas Sema7A^−/−^ mice showed a stronger increase in neutrophil infiltration in association with a prolonged resolution interval of up to 18 h (Fig. 4*A* and *SI Appendix*, Fig. S5). This response was affiliated with increases in the TNF-α, IL-6, and KC levels (Fig. 4*B*). When focusing on the specific features of the resolution phase, in which monocytes and MΦs predominantly control resolution programs, we observed a significant increase in classical Ly6C^hi^ monocytes and peritoneal MΦs and a strong decrease in alternatively activated Ly6C^lo^ monocytes. In this regard, the efferocytosis rate of apoptotic PMNs was strongly reduced in Sema7A^−/−^ mice (Fig. 4*C*), suggesting that Sema7A promoted an accelerated-resolution phenotype in acute peritonitis. Thus, endogenous SPMs derived from polyunsaturated fatty acids, e.g., lipoxins, resolvins, protectins, and maresins, play crucial roles in resolution programs. To elucidate the influence of Sema7A on the resolution processes, we developed a liquid chromatography-tandem mass spectrometry (LC-MS/MS)–based lipid-mediator profile (Fig. 4*D* and *SI Appendix*, Table S3) and observed significantly decreased LXA_4_ and PDX levels_._ This reduction was also reflected in metabolites and pathway markers such as 5-HETE, 18-HEPE, 14,15-diHETE, 7-HDHA, and 19,20d-DiHdPA. Next, we showed that Sema7A affected the repair and regeneration of peritoneal tissue, which resulted in a reduced proliferating cell nuclear antigen (PCNA) response in Sema7A^−/−^ mice compared with Sema7A^+/+^ mice (Fig. 4*E*). To more closely mimic sepsis, a life-threatening illness, we determined whether Sema7A could influence the mortality of mice afflicted with polymicrobial sepsis. We performed a survival test in a cecal ligation and puncture (CLP) model and found that Sema7A deficiency increased mortality rates and reduced survival by up to 78%. This effect was accompanied by a risk factor for increased mortality and hypothermia (Fig. 4*F*).

### Sema7A^SL4cd^ Fosters the Resolution of Acute Inflammation and Promotes Tissue Repair/Regeneration In Vivo.

**Fig. 5.**
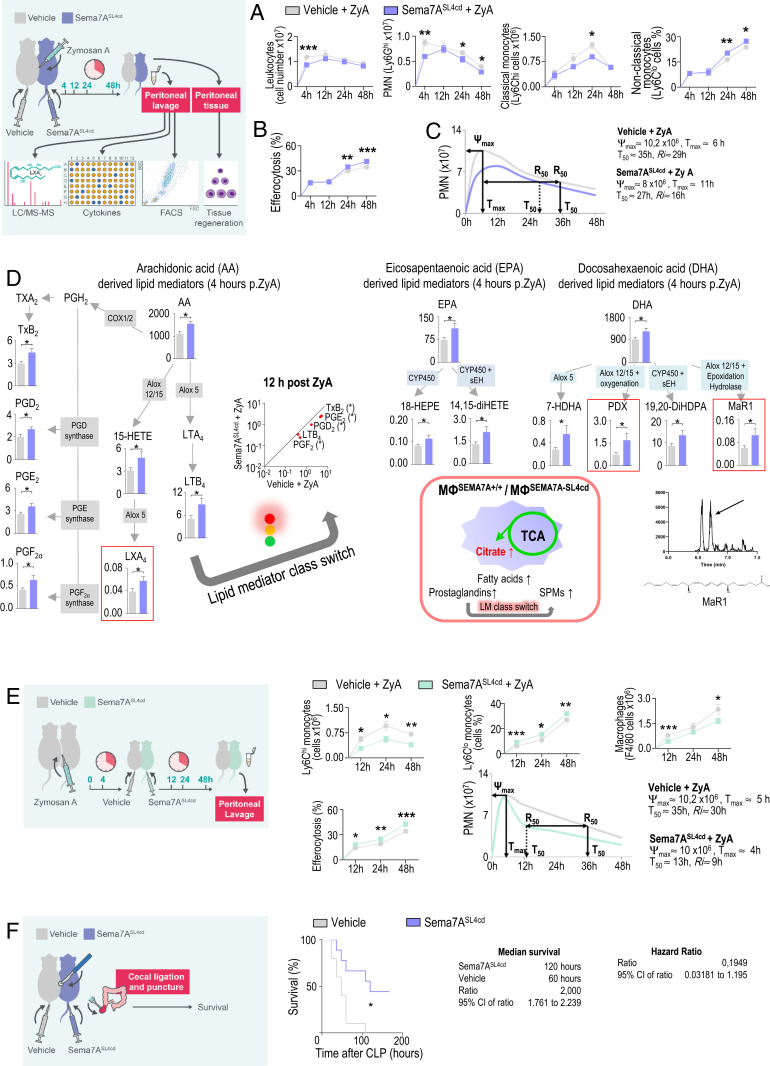
Exogenous Sema7A activates resolution programs. WT animals were sequentially injected with ZyA and vehicle or Sema7A^SL4cd^, and lavages were collected at 4, 12, 24, and 48 h. (*A*) Total leukocytes were counted by light microscopy, and PMNs and classical and nonclassical monocytes and (*B*) MΦ efferocytosis were assessed by flow cytometry (*n* = 6 to 17). (*C*) Resolution indices. (*D*) The levels of bioactive lipid mediators and precursors, including those in the AA, DHA, and EPA pathways, in the peritoneal fluids of WT animals that were treated with ZyA and Sema7A^SL4cd^ or vehicle for 4 or 12 h (*n* = 9 to 19) were quantified by LC-MS/MS–based profiling. (*E*) WT animals were injected with ZyA and treated with vehicle or Sema7A^SL4cd^ after 4 h at the peak of inflammation, and lavages were collected at 12, 24 and 48 h. Classical and nonclassical monocytes and MΦs as well as monocyte-derived macrophage efferocytosis were quantified by flow cytometry (*n* = 8 to 13). Resolution indices were determined. (*F*) The survival rates of WT animals treated daily with Sema7A^SL4cd^ or vehicle that underwent the CLP procedure and the median survival (*n* = 9 to 10). The results represent three independent experiments and are expressed as the mean ± SEM (*A*–*E*); significance was determined by the unpaired two-tailed Student’s *t* test (*A*, *B*, *D*, and *E*), and the log-rank test (*F*), *P < 0.05, **P < 0.01, ***P < 0.001.

We next focused on the potential therapeutic efficacy of Sema7A^SL4cd^ in acute inflammation. Using a ZyA-induced murine peritonitis model, the simultaneous administration of Sema7A^SL4cd^ and ZyA, reflecting prophylactic treatment, improved resolution effects, as demonstrated by reduced PMN recruitment, decreased Ly6C^hi^ monocytes, and increased Ly6C^lo^ monocytes within the peritoneal lavages (Fig. 5*A*). These features were accompanied by an increase in MΦ clearance and, ultimately, a shortening of the resolution interval from 29 to 16 h (Fig. 5 *B* and *C*). In Sema7A^SL4cd^ treated lavages, the levels of the inflammatory cytokines were significantly reduced at 4 h, whereas that of the proresolving/proregenerative cytokine TGF-β was significantly increased at 12 h, reflecting nonphlogistic attributes of Sema7A in cell recruitment (*SI Appendix*, Fig. S6 *A* and *B*). When performing PCNA immunohistochemical staining, we detected an increase in PCNA responses, implying that Sema7A contributes to tissue repair and regeneration processes (*SI Appendix*, Fig. S6*B*). The temporal regulation profile of Sema7A demonstrated that it was strongly expressed at 4 h and then gradually decreased in the resolution/regeneration phase (*SI Appendix*, Fig. S6*C*). When comparing this phenomenon with the lipid-mediator generation, Sema7A expression increased concomitantly with the induced generation of SPMs and their precursors and pathway markers (Fig. 5*D* and *SI Appendix*, Table S4). Furthermore, we found increased levels of PGD_2_ and PGE_2_ at 4 h after treatment with Sema7A^SL4cd^, whereas at 12 h, these levels were significantly reduced, suggesting that Sema7A led to a mediator class switch from prostaglandins and leukotrienes to the biosynthesis of proresolving lipid mediators. These findings substantiated our MΦ metabolomic data, where citrate was strongly expressed in Sema7A^+/+^, indicating increases in the production of lipids, prostaglandins, and, finally, SPMs (Fig. 4*D*). Next, we sought to evaluate the impact of Sema7A^SL4cd^ at the peak of inflammation, 4 h post ZyA treatment, to elucidate a potential therapeutic impact and found a stronger influence of Sema7A^SL4cd^ in resolution processes compared with the WT, resulting in a resolution interval of 9 vs. 30 h (Fig. 5*E* and *SI Appendix*, Fig. S6*D*). In a murine CLP sepsis model, Sema7A^SL4cd^ significantly decreased mortality rates and improved survival by up to 200% (Fig. 5*F*). We compared the impact of Sema7A^SL4cd^ with the full-length Sema7A protein at the peak of acute inflammation (4 h post ZyA). Even though the Sema7A protein showed a similar profile as Sema7A^SL4cd^ the effect of Sema7A^SL4cd^ is, however, stronger (*SI Appendix*, Fig. S6 *E* and *F*).

### Plasma Sema7A Is Associated with the Clinical Outcomes of Critically Ill Pediatric Patients with Abdominal Compartment Syndrome.

**Fig. 6.**
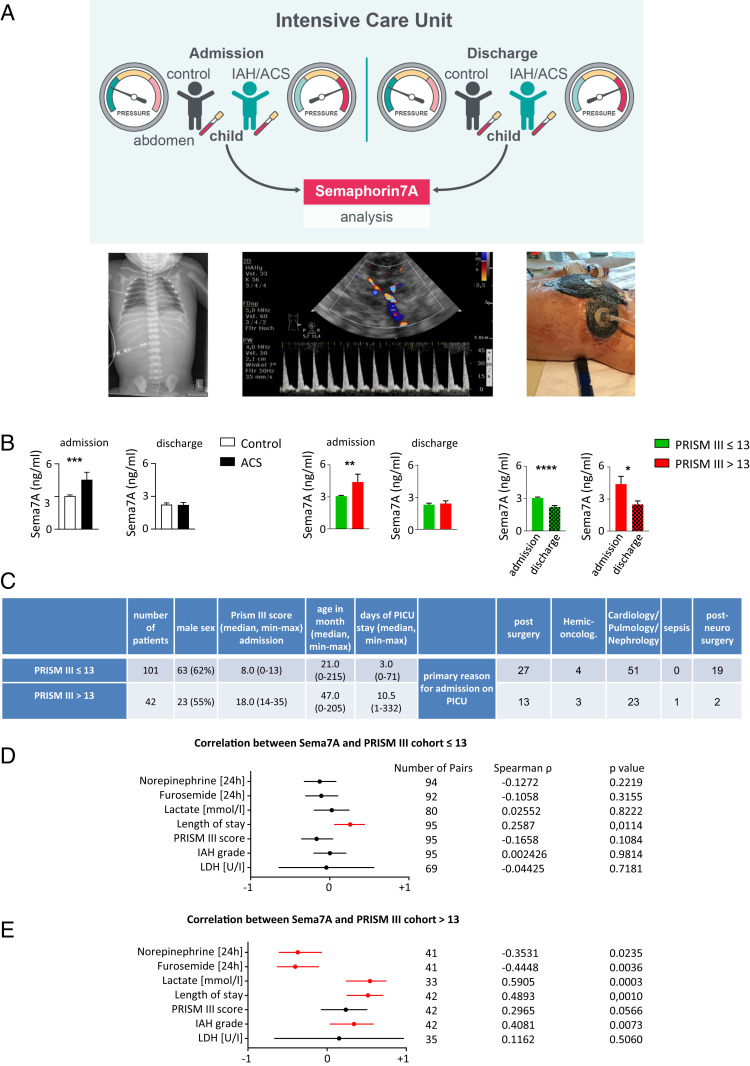
Sema7A in pediatric ICU patients with abdominal compartment syndrome. Plasma samples from 143 children in the ICU with and without ACS were collected within 24 h after admission and on the day of discharge from the PICU of Hannover Medical School. (*A*) Representative images displaying critically ill children with diaphragmatic elevation (*Left*), abdominal diastolic bloodflow in a patient with IAH (*Middle*) and a patient with ACS (after decompressive laparotomy with the establishment of an open abdomen/laparostoma to reduce intra-abdominal pressure and the associated tissue and organ impairments) (*Right*). (*B*) Sema7A levels were measured by ELISA on the day of admission to the ICU and on the day of discharge. (*C*) Overview of ICU patient characteristics with a PRISM score ≤13 and a PRISM score >13. (*D* and *E*) Correlation between Sema7A and the clinical parameters of patients in the PICU with PRISM III scores ≤13 (*D*) and >13 (*E*) on the day of admission. Spearman’s rank correlation coefficient Rho and the corresponding 95% CI interval are shown. The results are displayed as the mean ± SEM. CI; significance was determined by the nonparametric Mann–Whitney *U* test (*B*); correlation was assessed using the Spearman’s rank correlation test (*D* and *E*). *P < 0.05, **P < 0.01, ***P < 0.001, ****P < 0.0001.

In a cohort of 143 critically ill children suffering from abdominal compartment syndrome (ACS), ranging in age from newborns to <18 y, we determined a possible interdependence between the plasma Sema7A levels on the days of admission to and discharge from the pediatric intensive care unit (PICU) and the PRISM III score (Fig. 6 *A* and *B*) (20, 21). Vital signs, cardiorespiratory parameters, drug administration, fluid balance, and intraabdominal pressures were monitored constantly. Our data demonstrated strong alterations in plasma Sema7A levels in critically ill patients suffering from ACS during their stay in the PICU compared to those in the control group (Fig. 6*B*). Because of the inhomogeneity of illness severity, the subjects were then divided into two test groups, namely, those with PRISM III scores ≤13 and those with PRISM III scores >13 (Fig. 6*C*). Compared with the control group, critically ill children with PRISM III scores ≤13 displayed approximately three-fold higher Sema7A plasma concentrations, whereas critically ill children with PRISM III scores >13 demonstrated approximately five-fold higher levels on the day of admission. On the day of discharge, we observed a sharp decline in both groups to approximately the same level, suggesting that Sema7A acted as a progression marker, as it seemed to increase during the initial resolution processes and then decrease. The initial increase in Sema7A seems to provide a statement about the initial severity of the patient’s illness, implying that more severe disease is correlated with a higher Sema7A plasma concentration and, ultimately, with a stronger impact of intervening in resolution programs. These findings correlate with our murine peritonitis data showing that Sema7A expression was significantly increased at the peak of inflammation and subsequently decreased (*SI Appendix*, Fig. S6*C*), suggesting that Sema7A exhibits proresolving attributes, especially in the early resolution phase. When comparing the Sema7A concentrations with the clinically assessed parameters, we observed significantly increased correlation coefficients for the length of PICU stay in both groups (Fig. 6 *D* and *E*). Further significant correlations were found with the lactate level and intraabdominal hypertension (IAH) grade but only in critically ill patients with PRISM scores >13 (Fig. 6*E*). We did not find significant correlations with serum lactate dehydrogenase, suggesting that the increased Sema7A concentrations did not arise from cell lysis (Fig. 6 *D* and *E*).

## Discussion

Acute inflammation is an important component of protection against infection or injury. However, it is increasingly appreciated that nonresolving or sustained inflammation may lead to chronic changes within organs, resulting in severe critical illnesses such as peritonitis, sepsis, and respiratory distress syndrome (22). The underlying fundamental features of the nonresolving processes are still incompletely understood; however, the inflammatory response and host defense begin with the release of chemical mediators such as chemokines, cytokines, and lipid mediators to activate PMN recruitment within the affected tissue. This process is accompanied by monocyte/MΦ phagocytosis, leading to leukocyte clearance and the generation of local endogenous SPMs, termed the “resolution of inflammation,” which ultimately reverts the tissue to homeostasis (14, 23). Checkpoints exist during an inflammatory response to regulate the inappropriate progression of inflammation (24). Recent evidence has highlighted immunometabolism by which intracellular metabolic changes induce functional alterations in immune cells and, finally, in therapeutic potential (17). In response to environmental signals, such as danger signals and inflammatory mediators, cells of the monocyte/MΦ lineage can undergo metabolic changes with the emergence of different functional phenotypes (25).

NGPs were initially identified to be crucial for the development of the nervous system (3). Recent studies have revealed biological parallels and interactions between the nervous and immune systems, demonstrating crucial effects in inflammatory events (26–30). The class of semaphorins is being increasingly recognized as playing important roles in immune function (3). Sema7A is a GPI-linked protein and a cellular homolog of viral semaphorins encoded by vaccinia and herpesvirus (31). Following proteolytic cleavage, Sema7A can also exist as a soluble protein (5, 32, 33). Sema7A is expressed in a variety of immune and nonimmune cells and thus influences the immune response. Studies have demonstrated that Sema7A is crucial in T cell-, monocyte- and MΦ-mediated inflammatory responses in contact hypersensitivity, experimental autoimmune encephalomyelitis, and pulmonary fibrosis through interactions with either the α1β_1_ integrin or plexin C1 (32, 34). Recently, Sema7A was reported to regulate cytokine-induced memory-like responses in human natural killer cells, and erythrocyte-derived Sema7A was shown to induce thrombosis inflammation in myocardial ischemia/reperfusion injuries (33, 35). Emerging evidence has revealed that MΦ metabolism is extremely plastic and often reflects the pathologies associated with certain disease states (9). Until recently, these divergent metabolic reactions of MΦs were presumed to be controlled by environmental influences and local cytokine release. This approach was extended by the paradigm of M1 and M2 MΦs, which implies that MΦs in the ground state, M0, can be driven into either the M1 or M2 activation state after exposure to defined mediators (36). To allow an immune response, specialized cells of the immune system morph from a state of relative silence to a state of high activity (37). Although it has been reported that Sema7A is a negative regulator of MΦ activation and Il-10 expression (34), here we show that Sema7A differentiates and polarizes human MΦs toward the nonclassical M2 phenotype (an M2-like phenotype seemed rather unlikely), as demonstrated by the cell morphology and gene markers. This finding was substantiated by reductions in proinflammatory cytokines and an enhancement of IL-10, suggesting that Sema7A induced nonphlogistic cell recruitment, a key characteristic of the resolution programs (12). These properties of Sema7A are consistent with those observed by Kang et al. in regards to dextran sodium sulfate-induced colitis (6). Since the cells were stimulated with a Sema7A SL4cd peptide, a soluble protein sequence that possesses the RGD motif, our data show that Sema7A binds more specifically to the αvß1 integrin receptor and thus induces the M2 phenotype in both differentiation and polarization. Considering that innate immunity has been attributed independently of lymphocytes to adaptive function after initial insult, which is defined as “innate immune memory,” metabolic changes in cells that are involved in inflammation are crucial for its function (9). Classically activated MΦ, dendritic cells, and Th17 cells display a transition toward aerobic glycolysis, which is associated with the secretion of proinflammatory mediators and reactive oxygen species. On the other hand, cells that limit inflammation, such as alternatively activated MΦs, exhibit oxidative metabolism and antiinflammatory cytokine secretion (9, 38). Studies of the control of glycolytic and oxidative metabolic pathways have revealed that these metabolic programs importantly influence cellular inflammatory reactions and ultimately suggest a close relationship between metabolism and immunity. Our data reveal parallels in M1/M2 differentiation and polarization programs between epigenetic expression patterns and changes in cellular glycolysis/fatty acid oxidation equilibrium, suggesting that Sema7A is capable of both preconditioning and educating myeloid progenitors. Consistent with this, we identified major changes in Sema7A-deficient peritoneal MΦs, as reflected by enhanced levels of genes encoding key regulators, such as HK2, GAPDH, PGK1, and ENO1, which ultimately resulted in increased lactate production (1). Enhanced glycolysis was associated with induction of the PPP, which was specifically detected based on the increased expression of G6PD, PGLS, RPE, and TKT (16). Furthermore, the TCA cycle appeared to be disrupted at the IDH and succinate SDH steps, which resulted in increased levels of TCA cycle intermediates such as succinate and fumarate. An additional metabolic signature involving the induction of succinate as well as fumarate accumulation in MΦs^Sema7A−/−^ evolved from glutamine metabolism, which might be linked to trained immunity, showing that monocytes and MΦs are especially capable of undergoing long-term adaptation in inflammatory processes through epigenetic and metabolic reprogramming (18). However, given the data resulting from the specific deletion of Sema7A in peritoneal MΦs and the image stream, protein microarray, and NMR experiments, the predominant role of Sema7A in murine peritonitis appears to be mediated through the interaction with the αvß1 integrin receptor, resulting in activation of the mTOR and AKT1 phosphorylation signaling pathways within peritoneal MΦs, which are known to be critical for regulating MΦ metabolism and polarization toward the M2 phenotype (19). However, it must be considered that dynamic immune cell migration occurs during the resolution of inflammation, which might induce specific mechanisms to control the diverse cellular behaviors. A specific molecule/agent often has differential and diverse (e.g., contact-dependent, paracrine, or autocrine) effects on different types of target cells. While further studies are required, it is possible that Sema7A simultaneously activates different mechanisms in cells that contribute to resolution mechanisms, such as apoptotic neutrophils. The diversity of Sema7A is also reflected in nonimmune cells. Sema7A derived from erythrocytes is rapidly cleaved and, in its soluble form in plasma, promotes thrombus formation and thromboinflammatory myocardial damage through its direct interaction with GPIb (33). The authors speculate that Sema7A may modulate the mechanosensory activity of the receptor (33). Hong et al. demonstrated that Sema7A promotes endothelial-to-mesenchymal transition through ATF3-mediated TGF-β2/Smad signaling (39). Endothelial Sema7A up-regulates the transcription factor ATF3 by interacting with the β1 integrin, resulting in TGF-β2 transcription and activation of the TGF/Smad3-signaling pathway (39) .

As a part of the resolution phase, activated MΦs arise from both monocyte-derived MΦs, which are replenished from the bone marrow, and tissue-resident MΦs, a specific lineage derived from fetal stem cells (9). More recent research, however, postulates that different MΦ lineages are programmed to respond divergently to tissue injury and disease (9). Using a murine peritonitis model, we demonstrated that Sema7A induces an antiinflammatory and proresolving phenotype in not only monocyte-derived MΦs but also resident MΦs. This result suggests that Sema7A might have a strong impact on the resolution mechanisms. Nevertheless, it is highly important to better understand the functions of the specific MΦ lineages that affect the outcomes of inflammation or disease. Considering that no specific treatments are currently available that target severe systemic inflammatory diseases such as sepsis, investigations of the biological actions of immunoresolvents are crucial, as this approach is altered from "combating inflammation” to “focusing and promoting inflammation resolution” (38). While we cannot exclude the possibility that Sema7A might also have direct effects on the metabolism and polarization of other innate cells involved in resolution programs, our findings demonstrate that Sema7A affects five main resolution program components: 1) changing MΦ differentiation/polarization and MΦ metabolism toward the antiinflammatory and proresolving phenotypes; 2) damping PMN recruitment; 3) activating clearance; 4) releasing local endogenous proresolving mediators; and 5) shortening the resolution interval.

In conclusion, these results provide evidence that Sema7A is an immunoresolvent and indicate that NGPs have additional functions outside the central nervous system, potentially improving our understanding of inflammation resolution programs and their potential therapeutic value for the treatment of acute inflammatory diseases.

## Materials and Methods

Detailed descriptions of the complete material and methods are provided in *SI Appendix*.

### Animals.

This project was approved by the Institutional Review Board of Eberhard Karls Universität Tübingen and Regierungspräsidium Tübingen. C57BL/6 mice were purchased from Charles River Laboratories and used for the characterization of SEMA7A and exogenous application of the Sema7A^SL4cd^ peptide.

Semaphorin 7A knockout (*SEMA7A*^*−/−*^) mice on a *SvJ/129* background and littermate control mice (*SEMA7A*^*+/+*^) were bred and genotyped as previously described (5).

### Patients in the Pediatric ICU.

Critically ill children between 0 and 18 y of age were enrolled between January and August 2015 after written consent was obtained from their parents or guardians. The study was approved by the Local Ethics Committee of Medizinische Hochschule Hannover (MHH-No. 6677) and internationally registered (WHO-ICTRP DRKS00006556).

### Data Analysis.

The data were compared by one-way ANOVA or Student’s *t* test as appropriate. *P* values less than 0.05 were considered statistically significant.

## Supplementary Material

Supplementary File

Supplementary File

## Data Availability

All study data are included in the article and/or *SI Appendix*.
