# Supplementary material for: Sema7A is crucial for resolution of severe inflammation
Source: Proc Natl Acad Sci U S A. 2021 Feb 26;118(9):e2017527118. doi: 10.1073/pnas.2017527118 (PMC7936270; doi:10.1073/pnas.2017527118)
Supplement: Supplementary File [file pnas.2017527118.sapp.pdf]

PNAS
www.pnas.org

Supplementary Information for

Sema7A is Crucial for Resolution of Severe Inflammation

Andreas Körner[1], Alice Bernard[1], Julia C. Fitzgerald[2], Juan Carlos Alarcon-Barrera[3], Sarantos Kostidis[3], Torsten Kaussen[4], Martin Giera[3] and Valbona Mirakaj[1*]

[1]Department of Anesthesiology and Intensive Care Medicine, Molecular Intensive Care Medicine, University Hospital Eberhard-Karls University, Tübingen, Germany
[2]Hertie Institute for Clinical Brain Research, University Clinic Tübingen, Tübingen, Germany.
[3]Center for Proteomics and Metabolomics, Leiden University Medical Center (LUMC), The Netherlands
[4]Department of Pediatric Cardiology and Pediatric Intensive Care Medicine, Hannover Medical School, Hannover, Germany.

*Correspondence: Valbona Mirakaj, MD, PhD, Clinic of Anesthesiology and Intensive Care Medicine, Molecular Intensive Care Medicine, Eberhard-Karls University, Hoppe-Seyler-Straße 3, 72076 Tübingen

*E-mail: valbona.mirakaj@uni-tuebingen.de

SI Appendix table of contents:
Supplementary Information Text
Figures S1 to S6
Tables S1 to S3
Dataset S1
SI References

SI MATERIALS AND METHODS

SL4cd peptide. The SL4cd peptide CRGDQGGESSLSVSKWNTF is composed of 19 amino acids spanning positions 264 to 282 from murine SEMA7A (UniProt number Q9QUR8), and this region is evolutionarily conserved in humans (UniProt number O75326). The SL4cd peptide with an average mass of 2058.19 g/mol and theoretical isoelectric point of 6.21 was delivered as a lyophilized produce (Think peptides) and then reconstituted with ultrapure water to a concentration of 1 µg/µl.

Zymosan A Peritonitis. The animal protocols were used in accordance with the regulations of Regierungspräsidium Tübingen and the local ethics committee. The mice were intraperitoneally (i.p.) injected with 1 ml of ZyA (1 mg/ml; Sigma-Aldrich) and then with either vehicle or 1 µg of the SL4cd peptide in a total volume of 150 µl. Peritoneal fluids and tissues were obtained at 4, 12, 24 and 48 h and prepared as previously described (1).

Cecal ligation and puncture. The animal protocols were used in accordance with the regulations of Regierungspräsidium Tübingen and the local ethics committee. CLP procedure was performed as described before (2). Following the induction of anesthesia with ketamine/xylazine a longitudinal skin midline incision is done and linea alba is identified and dissected. Cecum is located and exteriorized by blunt forceps to prevent damage of the mesenterial blood vessels and intestine and then ligated 50%. The distal part of the cecum is then perforated with a 20-Gauge needle through-and-through. The cecum is relocated to the peritoneal cavity and peritoneum and skin closed with 5-0 sutures and the animals are resuscitated with 1 ml of prewarmed saline.

PMN and macrophage chemotaxis. MΦs were differentiated from peripheral blood monocytes (as described above). The microfluidic devices were printed using an S30L DLP printer (Rapidshape, Heimsheim, Germany) with photoresist MP300 (Rapidshape, Heimsheim, Germany). The device was constructed using Netfabb Professional 5.2 (Netfabb, Lupburg, Germany). Chemoattractant gradients, such as monocyte chemotactic protein (MCP-1) and SL4cd (± MCP-1), were established between a range of 8 peripheral chambers and a central cell loading well. The control chambers were loaded with RPMI medium. The cells were suspended at a density of 5×10^4 cells in a 10 µl total volume and placed into the central loading chamber. The cells were then incubated at 37°C for 8 h (MΦs) prior to imaging to allow generation of the chemotactic gradient. Cell migration was enumerated with a Casy TT cell counter (Omni Life Science, Bremen, Germany).

Human leukocyte isolation and MΦ differentiation and polarization. Human peripheral blood monocytes (PBMCs) were isolated from healthy volunteers or human leukapheresis collars from the Blood Bank of Eberhard Karls University of Tübingen. For *differentiation* experiments, cells were cultured in RPMI 1640 medium with 10 ng/ml human recombinant GM-CSF (Macs Milteny Bergisch Gladbach, Germany), 100 ng/ml M-CSF (Macs Milteny) or 1 µg of SL4cd peptide at 37°C for 7 d. In another set of experiments, M1 macrophages (cultured with GM-CSF for 7 d) were generated by stimulation with 100 ng/ml LPS (Sigma-Aldrich) and human recombinant IFN-γ (Promokine) ± 1 µg of Sema7A^{SL4cd} peptide for 2 d. M2 macrophages (cultured with M-CSF for 7 d) were generated by stimulation with human recombinant IL-4 (Promokine) and human recombinant IL-13 (R&D Systems, Inc) ± 1 µg of SL4cd peptide for 2 d. For *polarization*, M1 (cultured with GM-CSF for 7 d) macrophages were stimulated with 100 ng/ml of TNF-α (Promokine, Heidelberg, Germany) ± 1 µg of Sema7A^{SL4cd} peptide for 24 h, and then analysis was performed. Cell shape analysis was performed with

AxioVision software (AxioVision SE64 Rel. 4.9.1 Software, Carl Zeiss GmbH, Jena, Germany).

Analysis of monocyte/ MΦ differentiation and polarization, Sema7A. The transcriptional analysis of human *Sema7A* mRNA expression was performed using the sense primer 5'- CTC AGC ATC CAG CGA CAT -3' and antisense primer 5'- ACA GGG GCA CTA TCC ACA AG -3'. Human *18S* expression was evaluated with the sense primer 5'-GTA ACC CGT TGA ACC CCA TT-3' and antisense primer 5'- CCA TCC AAT CGG TAG TAG CG-3'. The following primers were used to determine the macrophage phenotype: *CD80*: 5'- AGC CTC ACC TCT CCT GGT TG-3', 5'- TGG GGC AAA GCA GTA GGT CA - 3'; *STAT-1:* 5'- ATC AGG CTC AGT CGG GGA ATA-3', 5'- TGG TCT CGT GTT CTC TGT TCT - 3'; *CD40* 5'- ACT GAA ACG GAA TGC CTT CCT-3', 5'- CCT CAC TCG TAC AGT GCC A - 3'; *Arg1*: 5'- TGG ACA GAC TAG GAA TTG GCA - 3', 5'- CCA GTC CGT CAA CAT CAA AAC T - 3'; *CD163*: 5' - ACA ACA GGT CGC TCA TCC C-3', 5'- GTG TGG CTC AGA ATG GCC T - 3'; *CD206*: 5'- CCC TCA GAA AGT GAT GTG CCT-3', 5'- TCT CCA CGA AGC CAT TTG GT - 3'; *IL-1β*: 5'- GAC CAC CAC TAC AGC AAG GG -3', 5'- ATC GTG CAC ATA AGC CTC GT - 3'; *IL-6*: 5'- CAC CAG GCA AGT CTC CTC AT - 3', 5'- GAC AGC CAC TCA CCT CTT CA - 3'; *IL-10*: 5'- AAT CGA TGA CAG CGC CGT AG - 3', 5'- GGT TGC CAA GCC TTG TCT GA - 3' and *TGFβ*: 5'- TGG TGG AAA CCC ACA ACG AA -3', 5'- GAA GTT GGC ATG GTA GCC CT -3'. The transcriptional analysis of the G protein-coupled receptors (GPCRs), such as ALX/FPR2 and GPR32, which have been shown to mediate pro-resolving actions, was performed using the following primers: *ALX/FPR2*: 5'- TGT TCT GCG GAT CCT CCC ATT- 3', 5'- CTC CCA TGG CCA TGG AGA CA- 3'. *GPR32:* 5'- GGG CCT GCA AAC TCT ACA - 3', 5'- GGA GGC AGT TAC TGG CAA - 3'.

The transcriptional analysis of murine Sema7A expression was performed using the following primers: *mu Sema7A*: 5'- GTG GGT ATG GGC TGC TTT TT - 3', 5'- CGT GTA TTC GCT

TGG TGA CAT - 3' and *mu* <u>*18S*</u>: 5'- GTA ACC CGT TGA ACC CCA TT -3', 5'- CCA TCC AAT CGG TAG TAG CG-3'.

For measuring protein levels, cells were blocked with Fc Receptor Blocking Solution (Biolegend) for 10 min at room temperature and then stained with anti-human CD40-e450 (Biolegend), CD163- FITC (Thermo Fisher), Arginase 1-PE (Biolegend), CD80-PerCP (Thermo Fisher), STAT1-PE (Biolegend) and CD206-APC (Biolegend) antibodies for 30 min at 4°C. The cells were analyzed by flow cytometry (BD FACSCanto II).

Cytology, immunofluorescent and immunohistochemical staining. MΦ that were differentiated from peripheral blood monocytes were labeled with Alexa Fluor 488-conjugated anti-SEMA7A (Santa Cruz Biotechnology) and anti-CD68 (Santa Cruz Biotechnology) antibodies. An IgG isotype control antibody (Santa Cruz Biotechnology) was used as a negative control. Alexa Fluor 488-conjugated goat Alexa Fluor 594-conjugated antibody (Life Technologies) was used as secondary antibody. DAPI (4',6-diamidino-2-phenylindole; Invitrogen) was employed for nuclear counterstaining. Immunofluorescence images were acquired using a confocal microscope (LSM 510 Meta fluorescence microscope, Carl Zeiss) and ZEN software (Carl Zeiss). To perform immunohistochemical staining for PCNA, FFPE peritoneal tissues were stained with an anti-PCNA antibody (Santa Cruz Biotechnology) using a Vectastain ABC Kit (Vector Labs) and DAB peroxidase substrate (Sigma-Aldrich) according to the manufacturers' instructions. As the secondary antibody, a biotin-conjugated rabbit-anti-mouse antibody (Jackson ImmunoResearch) was used. The sections were then counterstained with hematoxylin.

Human MΦ phagocytosis/efferocytosis. To prepare apoptotic PMNs, human PMNs obtained from peripheral blood were isolated and labeled with carboxyfluorescein diacetate (10 μM, 30 min at 37°C; Molecular Probes) and allowed to undergo apoptosis in serum-free RPMI 1640

medium for 16-18 h. GM-CSF-differentiated MΦ (0.1×10^6 cells/well) were then incubated with SL4cd peptide. Apoptotic PMNs were added at a 1:3 ratio (MΦ:PMN) and incubated at 37°C for 60 min to allow phagocytosis. In a separate set of experiments, GM-CSF-differentiated MΦ (0.1×10^6 cells/well) were incubated with SL4cd peptide, an anti-Plexin C1 antibody or siRNA targeting Integrins α1, αv and ß1 for 24 hours and fluorescent ZyA particles were added at a 1:10 ratio (MΦ:ZyA) and incubated at 37°C for 60 min to allow phagocytosis. IgG antibodies or scrambled siRNA were used as controls where appropriate. Fluorescence was determined using a fluorescent plate reader (Tecan, Männedorf, Switzerland).

Differential leukocyte counts, flow cytometry analysis and ELISA. Exudate cells from the murine peritonitis model were prepared to determine the cellular composition. The cells were blocked with mouse anti-CD16/CD32 antibodies for 10 min at room temperature and then stained with anti-mouse APC-Ly6G, e450-F4/80 (all from eBioscience) and FITC-Ly6C (BioLegend) antibodies for 30 min at 4°C. To analyze MΦ phagocytosis of apoptotic PMNs in vivo, the cells were permeabilized and then stained with PerCP-Cy5.5-conjugated anti-Ly6G (eBioscience) for 30 min at 4°C. The cells were analyzed by flow cytometry (BD FACSCanto II). Cytokines and Sema7A were measured in the murine peritoneal exudates by standard ELISA (R&D systems).

Pediatric ICU patient samples with and without Abdominal Compartment Syndrome (ACS). Plasma samples were obtained from 143 pediatric ICU patients from the Pediatric Intensive Care Unit (PICU) of Hannover Medical School (MHH, Germany) within 24 h after admission and at the day of discharge. The 2013 WSACS definitions (3) (with respect to IAP and ACS; www.wsacs.org) were used to define the abdominal compartment syndrome (ACS). The severity of illness in the ICU children was measured using PRISM III scoring(4). Vital and cardiorespiratory parameters (including ventilation parameters), drug administration, intra-

abdominal pressures (measured via the gastric Spiegelberg® monitoring system (5)) and fluid balances were recorded continuously via the digital patient data management system (mlife, mediside). A Sema7A ELISA (Cloud-Clone Corp., # SEB448Hu) was performed according to the manufacturer's instructions.

LC-MS/MS. Peritoneal lavage samples were spiked with 4 µL of an internal standard solution (containing PGE_4-d4, LTB_4-d4 15-HETE-d8 and DHA-d6 at a concentration of 50 ng/ml in methanol). The samples were transferred to a 12-ml glass vial, and 1.75 ml of methanol was added. The samples were centrifuged at 4,000 rpm for 5 min at 6°C, and the supernatant was transferred to a fresh 12-ml glass vial. The pellet was re-extracted with 500 µl of methanol and centrifuged as described above, and the organic extracts were combined. The methanol was partially removed under a gentle stream of nitrogen at 40°C for 30 min. The remaining methanolic extract (approximately 1.5 ml) was diluted with 8 ml of water, and 20 µl of 6 M HCl was added. The prepared samples were cleaned via solid phase extraction (SPE) (SepPak C18 200 mg, Waters, MA, USA). The samples were loaded onto preconditioned SPE cartridges (2 ml methanol, followed by 2 ml water), the cartridges were washed with 3 ml of water followed by 3 ml of *n*-hexane, and then the samples were eluted with 3 ml of methylformate. The eluate was dried under a gentle stream of nitrogen, reconstituted in 200 µL of 40% methanol, and injected.

LC-MS/MS analysis was performed as described below. Briefly, a QTrap 6500 mass spectrometer operating in negative ESI mode (Sciex, Nieuwerkerk aan den Ijssel, The Netherlands) was coupled to an LC system employing two LC-30AD pumps, a SIL-30AC autosampler, and a CTO-20AC column oven (Shimadzu, 's-Hertogenbosch, The Netherlands). A 1.7 µm Kinetex C_{18} 50 × 2.1 mm column protected with a C8 precolumn (Phenomenex, Utrecht, The Netherlands) was used, and the column was maintained at 50°C. A binary gradient of water (A) and MeOH (B) containing 0.01% acetic acid was generated as follows: 0 min 30%

B, held for 1 min, then ramped to 45% B at 1.1 min, 53.5% B at 2 min, 55.5% B at 4 min, 90% B at 7 min, and 100% B at 7.1 min, and held for 1.9 min. The injection volume was 40 µl, and the flow rate was 400 µl/min. For analyte identification, the mass transition used for each analyte was combined with its relative retention time (RRT). The calibration lines constructed with standard material for each analyte were used for quantification, and only peaks with a signal to noise (S/N) ratio > 10 were quantified.

Image Stream Multispectral Imaging Cytometry. In brief, human differentiated M1 macrophages were stimulated with 100 ng of TNF-α (Promokine, Heidelberg, Germany) for 24 hours and blocked with anti-CD16/CD32 antibodies for 10 min at room temperature and then stained with anti-human Alexa Fluor 647-CD49a, PE-CD51, PE/Cy5-CD29 (all from eBioscience) and FITC-SEMA7A (Invitrogen) antibodies for 30 min at 4°C. At least 10.000 cells were acquired for each sample with 40x magnification using the ImageStream mkII with the INSPIRE instrument controller software. (Luminex/amnis, USA). Data were analyzed with IDEAS Image analysis software (version 6.2). All samples were gated on viable single cells in focus. The feature Similarity was applied to identify Colocalization.

Metabolic Extracellular Flux Assay. In brief, peritoneal macrophages were isolated and plated in XF-96-cell culture plates ($1*10^6$ cells) (Seahorse Bioscience) and treated as indicated. Non-adherent cells were washed away once with PBS. To assess metabolic programs, Seahorse XF Glycolysis and Mito Stress Tests were carried out according to the manufacturer's instructions. ECARs and OCRs were measured in an XF-96 Flux Analyzer (Seahorse Bioscience). Changes in ECAR in response to glucose (final well concentration (fwc): 10 mM), OM (fwc: 1.0 µM), and 2-DG (fwc: 50 mM) injection were used to calculate glycolysis, and OXPHOS characteristics were calculated from changes in OCRs in response to OM (fwc: 1.0 µM), FCCP (fwc: 2.0 µM), and ROT + AA (fwc: 0.5 µM) injection. For normalization of the

results, cells were washed once with PBS, fixed with 4% PFA containing Hoechst 33342 for 5 to 10 min and subjected to fluorescence measurements using a fluorescence plate reader.

NMR spectroscopy and quantification of metabolites. NMR analysis of the intracellular metabolites was carried out as described(6). Briefly, the dried extracts were reconstituted in 250 μL of 0.15 M K_2HPO_4/KH_2PO_4 buffer (pH = 7.4) in 99.9% deuterated water (D2O), including 0.2 mM NaN_3 and 0.4 mM trimethylsilylpropionic acid sodium salt (TSP-d_4), and transferred to 3-mm NMR tubes.

NMR data were recorded on a 14.1 T NMR spectrometer (600 MHz for ^1H; Bruker Avance II) under standardized conditions for all samples. All spectra were processed for phase and baseline correction and referenced to TSP-d_4. One-dimensional (1D) spectra were imported into Chenomx NMR suit 8 (Chenomx Edmonton, Canada) for quantification. Metabolites were identified based on the Bbiorefcode (Bruker Biospin) and Chenomx databases. The structures of all annotated metabolites were then confirmed in 2D NMR experiments of the same samples. The concentrations of the quantified metabolites (mM) were normalized to the cell count per sample. The R packages ((http://www.r-project.org/, R version 3.4.4) "factoextra" was used for clustering and heatmap presentation of quantitative data.

Antibody Array for Protein Expression. Peritoneal monocytes/MΦ from Sema7A$^{-/-}$ and Sema7A$^{+/+}$ mice were used following 0 h and 4 h of ZyA-induced peritonitis. Protein and phosphorylation (mTOR Phospho Antibody Array, FullMoonBioscience, #PMT138) profiling of peritoneal monocytes/MΦ (pooled lavages from 4 mice / condition) was carried out according to the manufacturer's instructions. The images were acquired by the manufacturer. For each antibody, the average signal intensity of 6 replicates was normalized to the median signal of all antibodies on the array. The presented fold change represents the ratio of the normalized signal from Sema7A$^{-/-}$ compared with Sema7A$^{+/+}$ mice. GAPDH and beta actin

were used as housekeeping proteins. Data analysis was performed with IPA software (Qiagen). Pathways were substantiated and updated with the recent literature, KEGG database (HSA 04150; HSA 04151) and Reactome database (R-HSA-165159, R-HSA-198203).

Glucose Metabolism RT2 Profiler PCR Array. Peritoneal MΦ from Sema$^{-/-}$ and Sema7A$^{+/+}$ mice were used following 0 h and 4 h of ZyA treatment. RNA isolation and cDNA synthesis were carried out with the miRNeasy Mini Kit and RT2 Easy First Strand Kit (Qiagen). Expression profiling of peritoneal MΦ (pooled lavages from 2 mice / condition) was carried out according to the manufacturer's instructions. The presented fold change represents the ratio of the normalized signal from Sema$^{-/-}$ compared to Sema7A$^{+/+}$ mice. GAPDH, beta actin, beta-2 microglobulin, beta glucuronidase and heat shock protein 90 alpha were used as housekeeping proteins. Data analysis was performed using web-based RT2 Profiler PCR Array data analysis and IPA software (Qiagen). Pathways were substantiated and updated with the recent literature.

SI REFERENCES

1. Mirakaj V, Dalli J, Granja T, Rosenberger P, & Serhan CN (2014) Vagus nerve controls resolution and pro-resolving mediators of inflammation. *J Exp Med* 211(6):1037-1048.
2. Korner A*, et al.* (2018) Resolution of inflammation and sepsis survival are improved by dietary Omega-3 fatty acids. *Cell Death Differ* 25(2):421-431.
3. Kirkpatrick AW*, et al.* (2017) Update from the Abdominal Compartment Society (WSACS) on intra-abdominal hypertension and abdominal compartment syndrome: past, present, and future beyond Banff 2017. *Anaesthesiol Intensive Ther* 49(2):83-87.
4. Pollack MM*, et al.* (2016) The Pediatric Risk of Mortality Score: Update 2015. *Pediatr Crit Care Med* 17(1):2-9.
5. Malbrain ML, De laet I, Viaene D, Schoonheydt K, & Dits H (2008) In vitro validation of a novel method for continuous intra-abdominal pressure monitoring. *Intensive Care Med* 34(4):740-745.
6. Kostidis S, Addie RD, Morreau H, Mayboroda OA, & Giera M (2017) Quantitative NMR analysis of intra- and extracellular metabolism of mammalian cells: A tutorial. *Anal Chim Acta* 980:1-24.

A

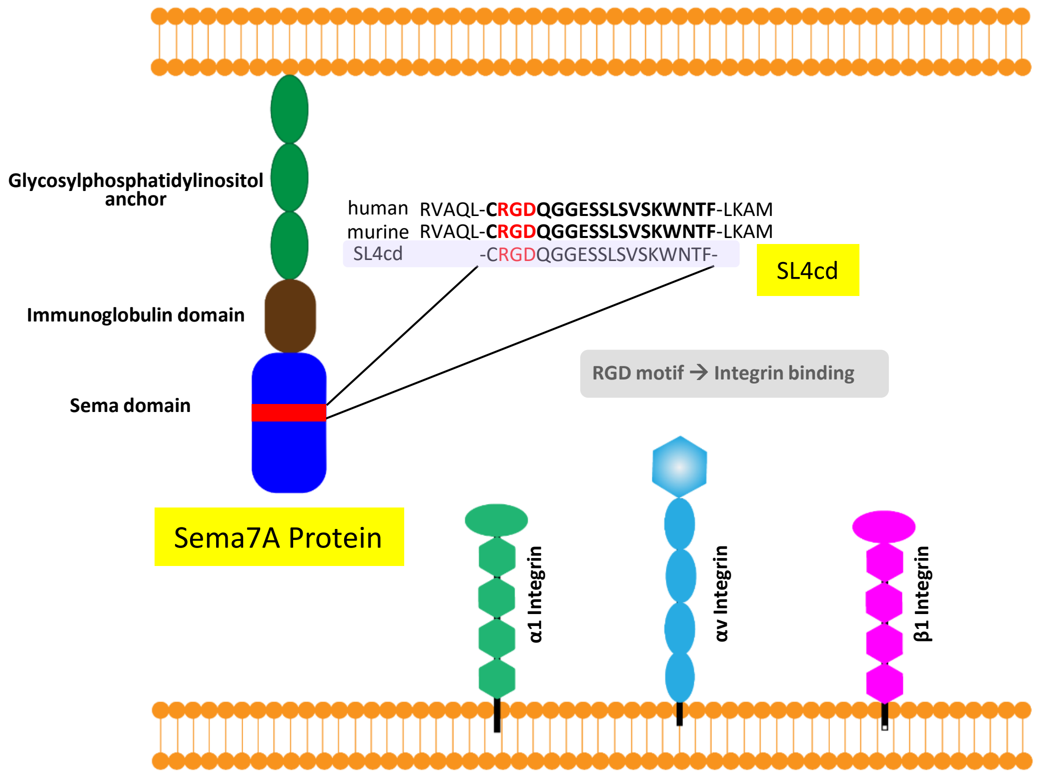

B

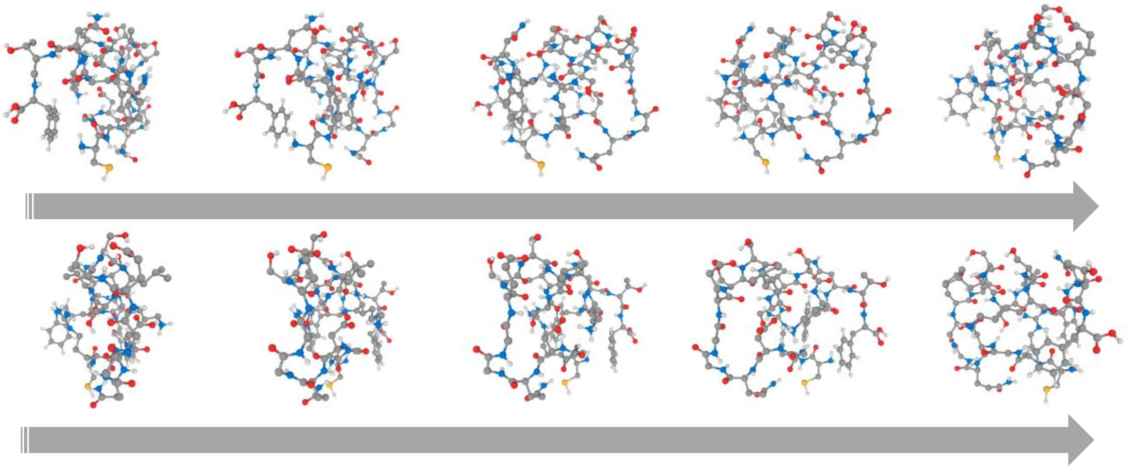

C

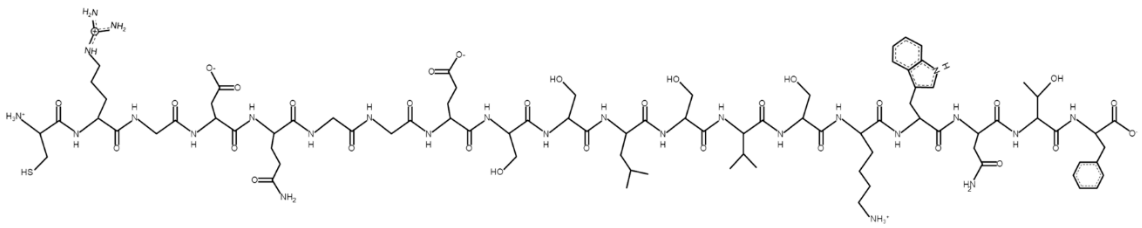

Figure S1

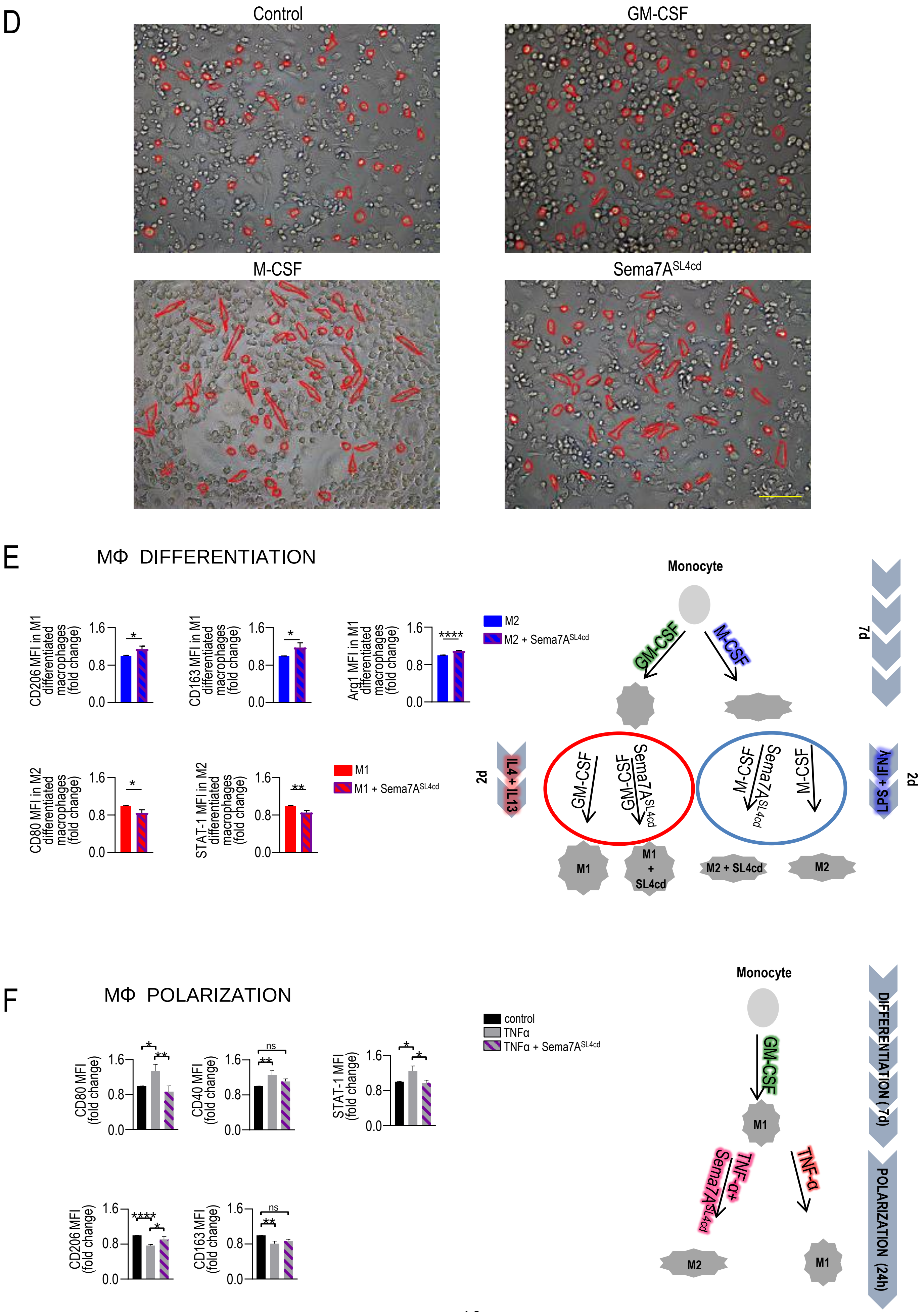
D
Control
GM-CSF
M-CSF
Sema7ASL4cd
E
MΦ DIFFERENTIATION
CD206 MFI in M1 differentiated macrophages (fold change)
CD163 MFI in M1 differentiated macrophages (fold change)
Arg1 MFI in M1 differentiated macrophage (fold change)
CD80 MFI in M2 differentiated macrophages (fold change)
STAT-1 MFI in M2 differentiated macrophages (fold change)
M2
M2 + Sema7ASL4cd
M1
M1 + Sema7ASL4cd
Monocyte
GM-CSF
M-CSF
7d
2d
IL4 + IL13
LPS + IFNγ
M1
M1 + SL4cd
M2 + SL4cd
M2
F
MΦ POLARIZATION
CD80 MFI (fold change)
CD40 MFI (fold change)
STAT-1 MFI (fold change)
CD206 MFI (fold change)
CD163 MFI (fold change)
control
TNFα
TNFα + Sema7ASL4cd
Monocyte
DIFFERENTIATION (7d)
POLARIZATION (24h)
TNF-α+ Sema7ASL4cd
TNF-α

Figure S1

Fig. S1: Sema7A controls macrophage inflammatory phenotype. A) Sema7A region and integrin receptors. **B)** Primary structure of the SL4cd peptide http://pepdraw.com/. **C)** Possible tertiary structure of the SL4cd peptide (http://bioserv.rpbs.univ-paris-diderot.fr/PEP-FOLD/). **D)** The cell morphology was analyzed by phase contrast images and measurements of the cell shape, cell length and perimeter (magnification ×200), Scale bar: 20µm. **E)** The expression of key markers that contribute to the M2 differentiation, CD206, CD163, and Arg1 and central genes of the M1 differentiation, STAT-1 and CD80, were analyzed (n=12). **F)** The expression of M1 polarization markers including CD80, CD40, STAT-1 as well as markers of the M2 polarization such as CD163 and CD206 were quantified by FACS analysis. The results are representative of 4 independent experiments and are expressed as the mean±SEM, unpaired two-tailed Student's t-test, *P< 0.05; **P< 0.01; ****P< 0.0001; ns= not significant.

A

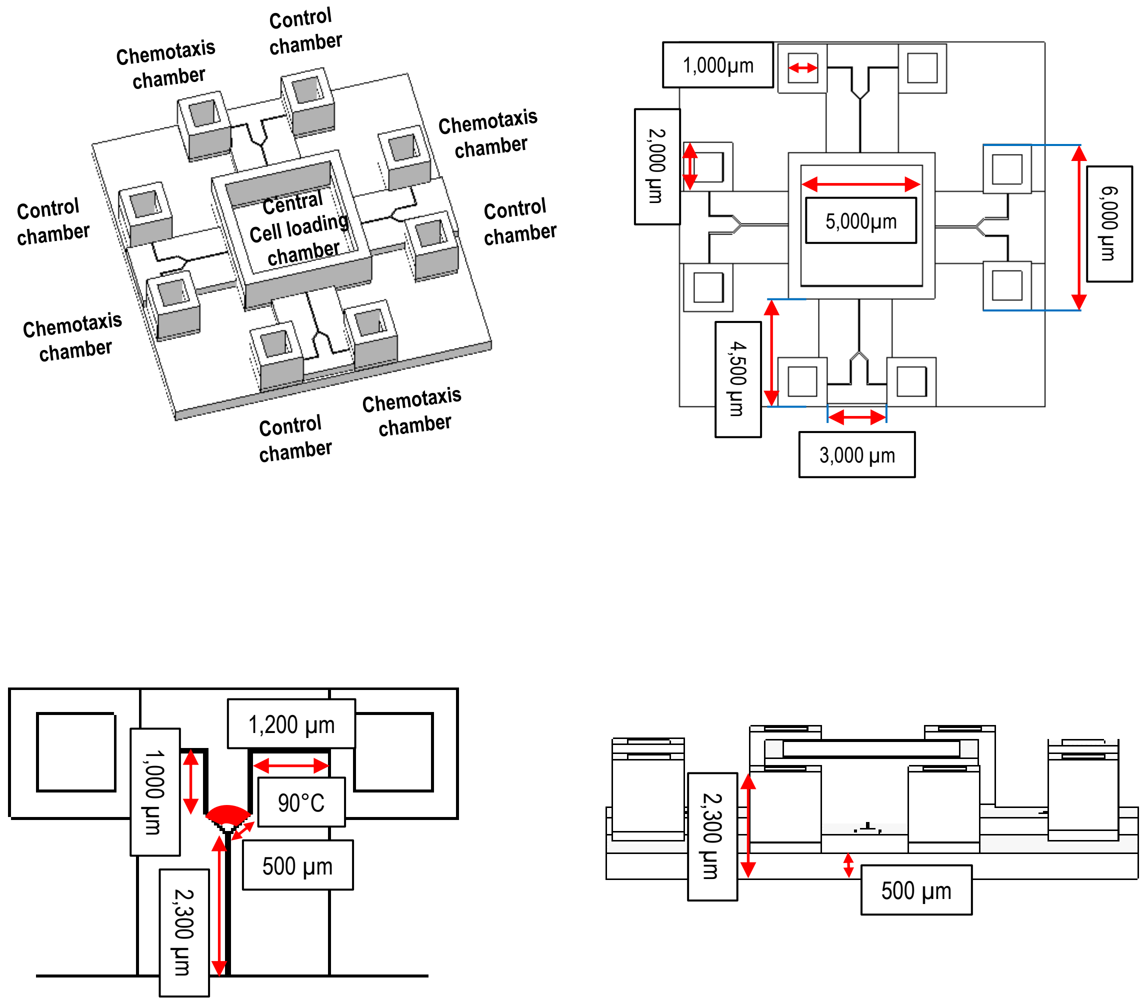
Control chamber
Chemotaxis chamber
Chemotaxis chamber
Control chamber
Control chamber
Central Cell loading chamber
Control chamber
Chemotaxis chamber
Chemotaxis chamber
Control chamber
1,000μm
2,000 μm
5,000μm
6,000 μm
4,500 μm
3,000 μm
1,200 μm
1,000 μm
90°C
500 μm
2,300 μm
2,300 μm
500 μm

Figure S2

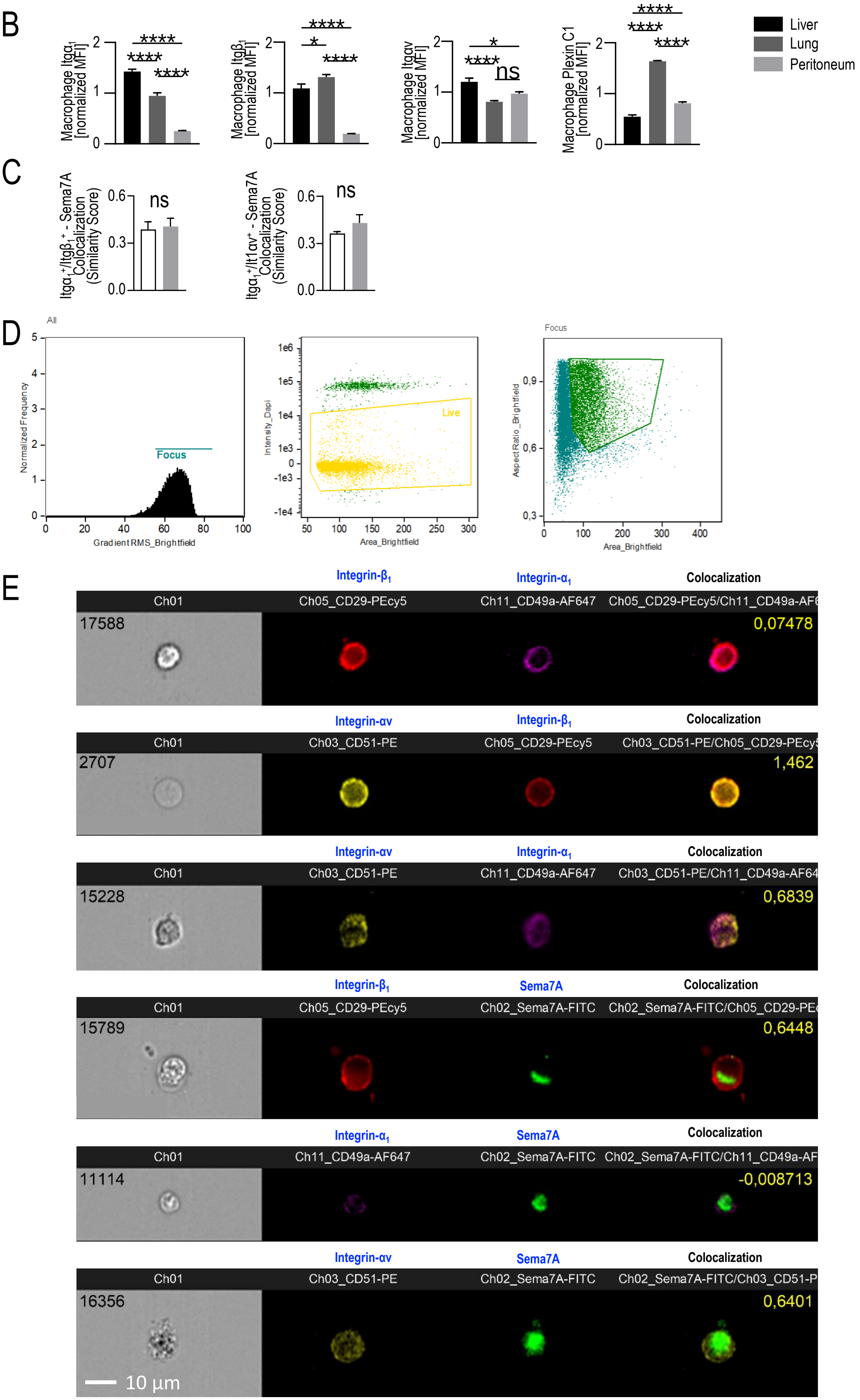
B
Liver
Lung
Peritoneum
Macrophage Itgα1 [normalized MFI]
Macrophage Itgβ1 [normalized MFI]
Macrophage Itgαv [normalized MFI]
Macrophage Plexin C1 [normalized MFI]
****
*
ns
C
Itgα1+/Itgβ1+ - Sema7A Colocalization (Similarity Score)
Itgα1+/It1αv+ - Sema7A Colocalization (Similarity Score)
ns
D
All
Normalized Frequency
Focus
Gradient RMS_Brightfield
Intensity_Dapi
Live
Area_Brightfield
Aspect Ratio_Brightfield
E
Integrin-β1
Integrin-α1
Integrin-αv
Sema7A
Colocalization
Ch01
Ch05_CD29-PEcy5
Ch11_CD49a-AF647
Ch03_CD51-PE
Ch02_Sema7A-FITC
17588
0,07478
2707
1,462
15228
0,6839
15789
0,6448
11114
-0,008713
16356
0,6401
10 µm

Figure S2

Fig. S2: Sema7A controls M1 MΦ chemotaxis and chemokinesis and clearance via interacting with integrin receptors. **A)** Large-scale scheme and fabrication of the microfluidic device. (Magnification 5×). Micro fluidic devices containing eight peripheral and one central cell-loading chamber were printed using an S30L DLP printer (Rapidshape, Heimsheim, Germany) with photoresist MP300 (Rapidshape) according to the manufacturer's instructions. The device was constructed using Netfabb Professional 5.2 (Netfabb, Lupburg, Germany). Chemotaxis and chemokinesis measurement: MΦ were stained with 10 µl of 0,05% rhodamine-6G (per 1 ml of medium; Sigma-Aldrich) according to the manufacturer's instructions. First, to ensure fluidic communication between all channels, the peripheral chemotaxis chambers were loaded with 1 µl of RPMI and the central cell-loading chamber was loaded with 25 µl of RPMI. To establish the chemoattractive gradients, the chemotaxis chambers were loaded with MCP-1 (1 µl; R&D Systems) or the Sema7A^{SL4cd} (1 µl), whereas the control chambers were loaded with RPMI to assess the impact on the chemokinesis response. Ten microliters of cell solution (containing $5x10^4$ cells) were added to the central cell-loading chamber. The cells were incubated at 37°C for 8 h (macrophages) prior enumerating the recruited cells using a Casy TT cell counter (Omni Life Science, Bremen, Germany). **B)** The expression of integrin receptors Itgα_1, Itgαv and Itgβ_1 was analyzed in Kupffer cells, alveolar and peritoneal macrophages. **C)** Colocalization of Itgα_1^+/Itgβ_1^+ or Itgα_1^+/Itgαv$^+$ cells with Sema7A. **D)** Gating strategy and **E)** example immunofluorescent pictures of analyzed monocytes/macrophages using Image Stream Multispectral Imaging Cytometry. The results are representative of 2 independent experiments and are expressed as the mean±SEM, unpaired two-tailed Student's t-test, *P< 0.05; ****P< 0.0001; ns= not significant.

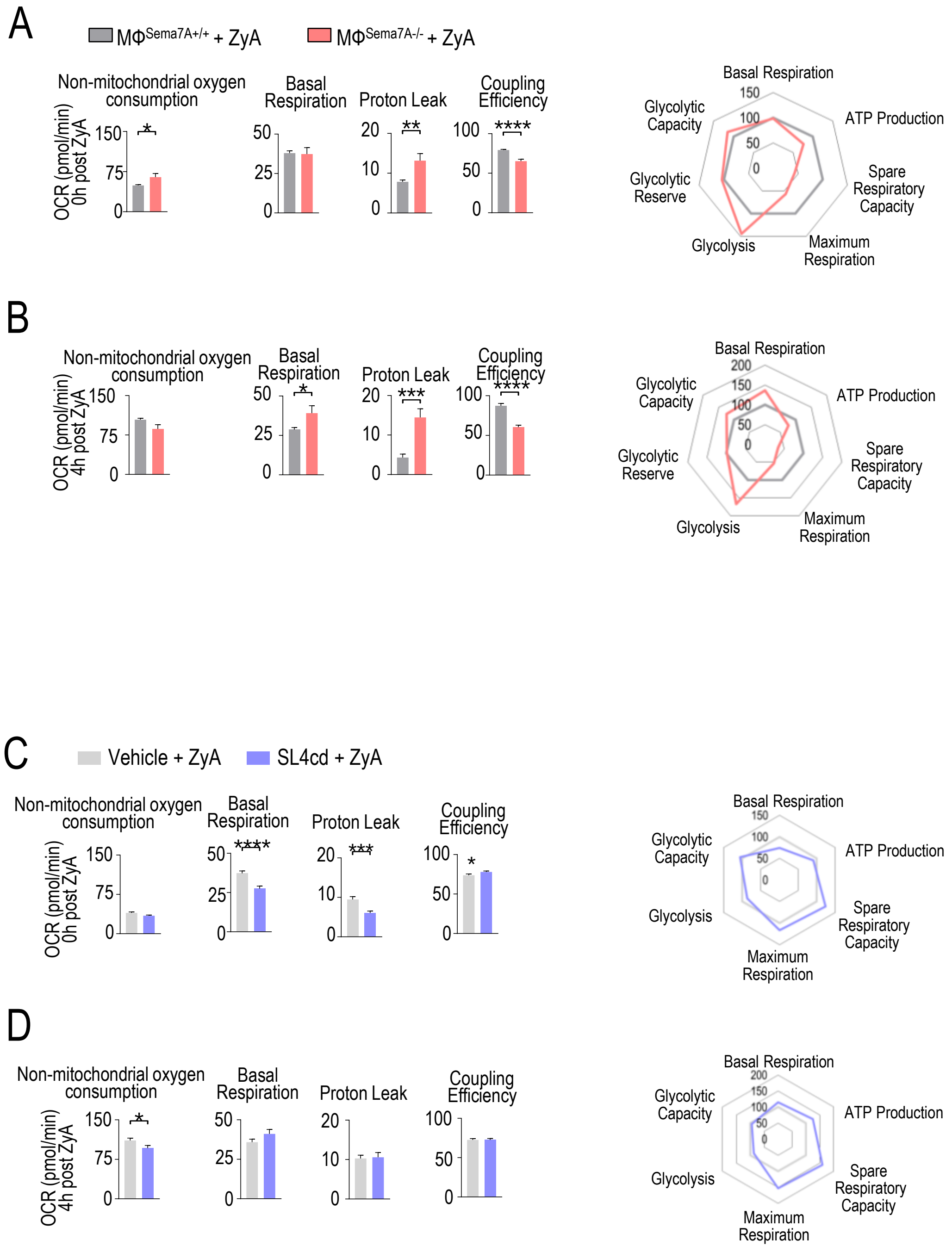
A
MΦSema7A+/+ + ZyA
MΦSema7A-/- + ZyA
Non-mitochondrial oxygen consumption
Basal Respiration
Proton Leak
Coupling Efficiency
OCR (pmol/min) 0h post ZyA
150
75
0
50
25
0
20
10
0
100
50
0
*
**
****
Basal Respiration
ATP Production
Spare Respiratory Capacity
Maximum Respiration
Glycolysis
Glycolytic Reserve
Glycolytic Capacity
150
100
50
0
B
Non-mitochondrial oxygen consumption
Basal Respiration
Proton Leak
Coupling Efficiency
OCR (pmol/min) 4h post ZyA
*
***
****
Basal Respiration
ATP Production
Spare Respiratory Capacity
Maximum Respiration
Glycolysis
Glycolytic Reserve
Glycolytic Capacity
200
150
100
50
0
C
Vehicle + ZyA
SL4cd + ZyA
Non-mitochondrial oxygen consumption
Basal Respiration
Proton Leak
Coupling Efficiency
OCR (pmol/min) 0h post ZyA
****
***
*
Basal Respiration
ATP Production
Spare Respiratory Capacity
Maximum Respiration
Glycolysis
Glycolytic Capacity
D
Non-mitochondrial oxygen consumption
Basal Respiration
Proton Leak
Coupling Efficiency
OCR (pmol/min) 4h post ZyA
*
Basal Respiration
ATP Production
Spare Respiratory Capacity
Maximum Respiration
Glycolysis
Glycolytic Capacity

Figure S3

Fig. S3: Glycolytic and mitochondrial respiration markers. Markers were calculated at **A)** baseline and **B)** following stimulation with ZyA for 4 h in $MΦ^{SEMA7A+/+}$ and $MΦ^{SEMA7A-/-}$. ECAR and OCR were also determined in MΦ from C57/BL/6 mice and either stimulated with vehicle or $Sema7A^{SL4cd}$ at **C)** baseline and **D)** following stimulation with ZyA for 4 h (n=20). The results represent three independent experiments and are expressed as the mean±SEM, unpaired two-tailed Student'st-test, *P<0.05; **P<0.01; ***P<0.001; ****P<0.0001.

		0h		4h	
Common name	Pubchem ID	Fold Regulation	P-value	Fold Regulation	P-value
Acly	Mm.282039	1,05	0,735663	-1,18	0,608829
Aco1	Mm.331547	2,21	0,199008	-1,06	0,990458
Aco2	Mm.154581	1,46	0,810875	1,15	0,784023
Agl	Mm.237099	1,53	0,566701	1,47	0,993239
Aldoa	Mm.275831	1,30	0,81027	1,69	0,507505
Aldob	Mm.482116	-1,56	0,672081	-2,23	0,91976
Aldoc	Mm.7729	-1,49	0,67004	1,86	0,348859
Bpgm	Mm.282863	1,74	0,303719	1,04	0,896477
Cs	Mm.58836	2,00	0,048261	2,01	0,498818
Dlat	Mm.285076	1,39	0,925942	-1,11	0,556708
Dld	Mm.3131	1,30	0,726659	1,49	0,686866
Dlst	Mm.296221	1,34	0,817908	1,18	0,735715
Eno1	Mm.372389	3,40	0,129529	1,18	0,499325
Eno2	Mm.3913	-1,32	0,662102	2,34	0,285012
Eno3	Mm.251322	1,39	0,233033	2,76	0,716661
Fbp1	Mm.423078	-1,52	0,712575	-1,66	0,827105
Fbp2	Mm.391871	-1,56	0,672081	-2,79	0,254622
Fh1	Mm.41502	1,09	0,675361	-1,01	0,927316
G6pc	Mm.18064	-1,56	0,672081	4,93	0,377184
G6pc3	Mm.22385	1,37	0,961281	2,45	0,525686
G6pdx	Mm.27210	1,49	0,736104	4,85	0,341391
Galm	Mm.29098	-1,44	0,773431	2,10	0,063496
Gapdhs	Mm.436562	-1,56	0,672081	1,68	0,627247
Gbe1	Mm.396102	-1,42	0,814887	-1,21	0,62135
Gck	Mm.220358	-1,56	0,672081	-2,79	0,254622
Gpi1	Mm.589	1,75	0,493424	2,32	0,354947
Gsk3a	Mm.491101	1,77	0,329699	5,44	0,320706
Gsk3b	Mm.394930	-1,55	0,975231	-2,16	0,339972
Gys1	Mm.275654	2,01	0,025336	5,34	0,246515
Gys2	Mm.275975	-1,56	0,672081	-2,79	0,254622
H6pd	Mm.22183	1,64	0,415361	1,80	0,304068
Hk2	Mm.255848	-1,23	0,771036	1,14	0,979639
Hk3	Mm.267479	1,13	0,877034	-1,09	0,674309
Idh1	Mm.9925	-1,46	0,491943	1,44	0,93756
Idh2	Mm.246432	1,64	0,262437	2,19	0,263296
Idh3a	Mm.279195	1,63	0,940981	3,34	0,3696
Idh3b	Mm.29590	1,44	0,841976	1,83	0,498517
Idh3g	Mm.14825	1,33	0,927357	1,78	0,408557
Mdh1	Mm.212703	1,85	0,381774	1,48	0,465855
Mdh1b	Mm.30494	-1,56	0,672081	-1,36	0,916599
Mdh2	Mm.297096	1,10	0,778669	1,07	0,769796
Ogdh	Mm.276348	2,04	0,494537	2,03	0,300017
Pck1	Mm.266867	-1,56	0,672081	-2,20	0,304983
Pck2	Mm.29856	2,29	0,346785	3,07	0,262542
Pcx	Mm.1845	-1,56	0,672081	1,45	0,282535
Pdha1	Mm.34775	1,50	0,540804	1,22	0,922865
Pdhb	Mm.301527	1,02	0,813497	-1,07	0,962043
Pdk1	Mm.34411	1,37	0,510909	3,38	0,220801
Pdk2	Mm.29768	1,67	0,34786	3,76	0,333413
Pdk3	Mm.12775	1,34	0,434956	1,25	0,741508

Table S1

		0h		4h	
Common name	Pubchem ID	Fold Regulation	P-value	Fold Regulation	P-value
Pdk4	Mm.235547	-1,38	0,665091	2,52	0,136573
Pdp2	Mm.290750	1,92	0,253826	1,25	0,76812
Pdpr	Mm.370024	1,33	0,164603	4,18	0,30667
Pfkl	Mm.269649	2,58	0,013193	1,39	0,945751
Pgam2	Mm.219627	-1,55	0,671867	1,23	0,325414
Pgk1	Mm.316355	1,27	0,585058	1,06	0,925913
Pgk2	Mm.717	-1,22	0,655301	-4,47	0,36119
Pgm1	Mm.2325	1,08	0,864225	1,49	0,339103
Pgm2	Mm.217764	1,88	0,383126	1,53	0,410848
Pgm3	Mm.390201	-1,28	0,882022	1,79	0,887823
Phka1	Mm.212889	1,62	0,332511	1,38	0,583665
Phkb	Mm.237296	1,78	0,125284	3,45	0,410028
Phkg1	Mm.3159	-1,41	0,666739	-2,31	0,481575
Phkg2	Mm.274473	1,58	0,606331	4,64	0,108417
Pklr	Mm.383180	-1,56	0,672081	-1,96	0,279366
Prps1	Mm.287178	1,12	0,922877	1,11	0,825032
Prps1l1	Mm.79179	-1,56	0,672081	1,22	0,730232
Prps2	Mm.272955	1,16	0,602751	1,63	0,305174
Pygl	Mm.256926	-1,81	0,471926	-1,51	0,423704
Pygm	Mm.27806	1,01	0,69256	1,39	0,633565
Rbks	Mm.22519	-1,10	0,836829	1,16	0,972425
Rpe	Mm.240912	1,01	0,923071	1,44	0,726419
Rpia	Mm.17905	-1,15	0,702006	-1,66	0,496704
Sdha	Mm.158231	1,28	0,571974	1,81	0,268833
Sdhb	Mm.246965	1,04	0,845746	1,39	0,58791
Sdhc	Mm.198138	1,50	0,900851	1,95	0,406175
Sdhd	Mm.10406	1,19	0,522771	2,48	0,121322
Sucla2	Mm.38951	1,16	0,991268	1,08	0,945351
Suclg1	Mm.29845	1,26	0,978268	2,93	0,344079
Suclg2	Mm.426727	-1,01	0,765637	2,61	0,276047
Taldo1	Mm.29182	-1,67	0,395366	-1,25	0,469803
Tkt	Mm.290692	1,53	0,832119	1,61	0,458042
Tpi1	Mm.4222	2,46	0,214087	1,14	0,792374
Ugp2	Mm.28877	-1,04	0,669493	1,43	0,762804
Actb	Mm.328431	2,32	0,497011	3,21	0,230132
B2m	Mm.163	1,08	0,583793	1,20	0,924851
Gapdh	Mm.309092	2,27	0,501002	1,49	0,416577
Gusb	Mm.3317	1,00	0	1,00	0
Hsp90ab1	Mm.2180	1,44	0,906627	1,63	0,523509

Table S1

Table S1: Glycolytic enzyme expression fold regulation in MΦ$^{SEMA7A+/+}$ and MΦ$^{SEMA7A-/-}$ evaluated by PCR array at baseline and 4 h post ZyA. Results represent three independent experiments and are presented in fold change values (MΦ$^{SEMA+/+}$ / MΦ$^{SEMA+/+}$).

	0h					4h				
	SEMA7A$^{+/+}$		SEMA7A$^{-/-}$			SEMA7A$^{+/+}$		SEMA7A$^{-/-}$		
	MEAN	S.E.M	MEAN	S.E.M	P-value	MEAN	S.E.M	MEAN	S.E.M	P-value
Lactate	30,1	5,021	53,63	0,325	0,0361	41,26	3,819	47,47	3,365	0,2899
ATP	2,24	0,148	3,567	0,2603	0,0114	1,757	0,2298	2,517	0,1167	0,042
Acetate	0,98	0,0781	1,567	0,1167	0,0139	0,9	0,06083	1,35	0,0866	0,0131
Alanine	6,737	0,944	10,48	0,7812	0,0377	9,777	0,4067	16,08	0,6333	0,0011
Arginine	3,597	0,3998	6,15	0,5204	0,0177	8,403	0,6094	10,88	0,4177	0,0284
Asparagine	1,313	0,2034	2,183	0,2242	0,0453	1,99	0,03606	3,133	0,1453	0,0016
Aspartate	0,89	0,06557	1,583	0,06667	0,0018	3,86	0,1493	3,2	0,07638	0,017
Choline	0,2	0,01	0,2833	0,01667	0,0128	0,08	0,01	0,1667	0,01667	0,0112
Citrate	1,06	0,06245	0,9333	0,1302	0,4298	1,89	0,1609	1,767	0,1202	0,5724
Citrulline	0,32	0,05568	0,5667	0,2167	0,3321	4,537	0,1849	6,083	0,2522	0,0078
Creatine	4,007	0,3227	6,033	0,6366	0,0469	4,647	0,2185	5,85	0,2843	0,0284
Creatine phosphate	4,163	0,3982	4,8	0,1732	0,2165	4,04	0,05568	5,167	0,2949	0,0199
Cysteine						1,203	0,181	1,85	0,05	0,0262
Ethanol	1,013	0,3044	1,667	0,0928	0,1093	0,9633	0,2849	1,717	0,8007	0,4255
Formate	1	0,1082	1,6	0,07638	0,0106	1,153	0,2243	1,517	0,1167	0,2241
Fumarate	0,13	0,02	0,2667	0,01667	0,0063	0,44	0,02646	0,55	0,02887	0,0484
Glucose	9,69	1,728	13,95	3,453	0,3318	5,08	0,5941	14,6	1,851	0,0081
Glutamate	12,7	1,1	18	0,9042	0,0204	11,19	0,3379	14,4	0,522	0,0067
Glutamine	4,06	0,5877	5,65	0,7077	0,159	6,02	0,197	9,85	0,293	0,0004
Glutathione	6,607	0,8093	9,417	0,5674	0,0467	6,01	0,3201	6,867	0,2455	0,1009
Glycine	1,12	0,148	1,7	0,1	0,0315	1,27	0,0755	1,783	0,1167	0,0209
Guanidinosuccinate						1,41	0,03606	1,533	0,0441	0,0963
Guanidoacetate	0,24	0,01732	0,3333	0,03333	0,0679	0,22	0,01	0,3167	0,01667	0,0076
Histidine						0,42	0,03	0,6	0,02887	0,0124
dihydroxyacetone phosphate						0,4967	0,0393	0,7833	0,01667	0,0026
Hypotaurine	6,727	0,5899	10,07	0,9112	0,037	4,687	0,3756	5,767	0,159	0,0571
Isoleucine	0,96	0,1908	1,317	0,1878	0,2536	1,29	0,02646	2,233	0,174	0,0058
Isopropanol	0,32	0,05568	0,7	0,05	0,0071	0,33	0,0755	0,6333	0,2048	0,237
Itaconic acid	1,26	0,09849	2,033	0,1364	0,0101	0,94	0	0,9667	0,01667	0,1848
Leucine	0,86	0,197	1,183	0,1764	0,2885	1,3	0,05196	2,2	0,2021	0,0125
Lysine	0,6567	0,126	0,9	0,07638	0,174	1,79	0,1212	2,167	0,1364	0,108
Malate	1,12	0,1873	2,533	0,2242	0,0084	4,02	0,1345	5,517	0,348	0,016
Methionine	0,28	0,04359	0,4	0,05	0,1447	0,4	0,02646	0,6833	0,0441	0,0053
NAD+	1,23	0,1153	1,717	0,1453	0,0586	0,74	0,05	0,95	0,07638	0,0829
O-Acetylcarnitine	0,08	0,01	0,08333	0,01667	0,8722	0,03	0	0,03333	0,01667	0,8512
O-Phosphocholine	1,433	0,1009	2,133	0,159	0,0205	1,03	0,01732	1,333	0,03333	0,0013
O-Phosphoethanolamine	3,767	0,1695	6,9	0,5008	0,0041	5,493	0,2195	8,017	0,5667	0,0142
Ornithine	0,24	0,03	0,2667	0,0441	0,6433	0,5267	0,05333	0,3667	0,08333	0,1812
Proline	0,6367	0,1789	1,1	0,2021	0,1612	1,19	0,1114	1,667	0,2167	0,122
Putrescine						0,62	0,04	0,7833	0,0441	0,0517
Serine	0,79	0,1082	1,25	0,07638	0,0255	1,19	0,1114	2,25	0,1	0,0021
Succinate	0,7	0,09644	0,7333	0,08819	0,8112	0,35	0,02646	0,4	0	0,1318
Taurine	26,57	1,875	39,17	2,584	0,0169	18,27	0,9368	23,2	1,29	0,0366
Threonine	0,34	0,02646	0,6667	0,0441	0,0031	0,4967	0,1433	1,1	0,1041	0,0271
Tyrosine	0,35	0,06083	0,4	0,07638	0,6356	0,4533	0,06333	0,7	0,02887	0,0239
Valine	0,4733	0,07311	0,7333	0,1014	0,106	0,63	0,03606	1,15	0,0866	0,0052
myo-Inositol	1,02	0,04	1,6	0,1155	0,009	1,23	0,04359	1,8	0,02887	0,0004
sn-Glycero-3-phosphocholine	3,143	0,2288	3,983	0,0928	0,0272	14,02	0,4665	20,37	0,9329	0,0037
beta-Alanine	2,16	0,1819	3,167	0,3632	0,0684	1,437	0,1475	1,567	0,1014	0,5078

Table S2

Table S2: Intracellular metabolite fold regulation evaluated by NMR analysis in $M\Phi^{SEMA7A+/+}$ and $M\Phi^{SEMA7A-/-}$ at baseline and following stimulation with ZyA 4 h. All results are reported in micromolar per million cells. Results represent one experiment with n=3 mice/group and are expressed as the mean±SEM, unpaired two-tailed Student'st-test.

A

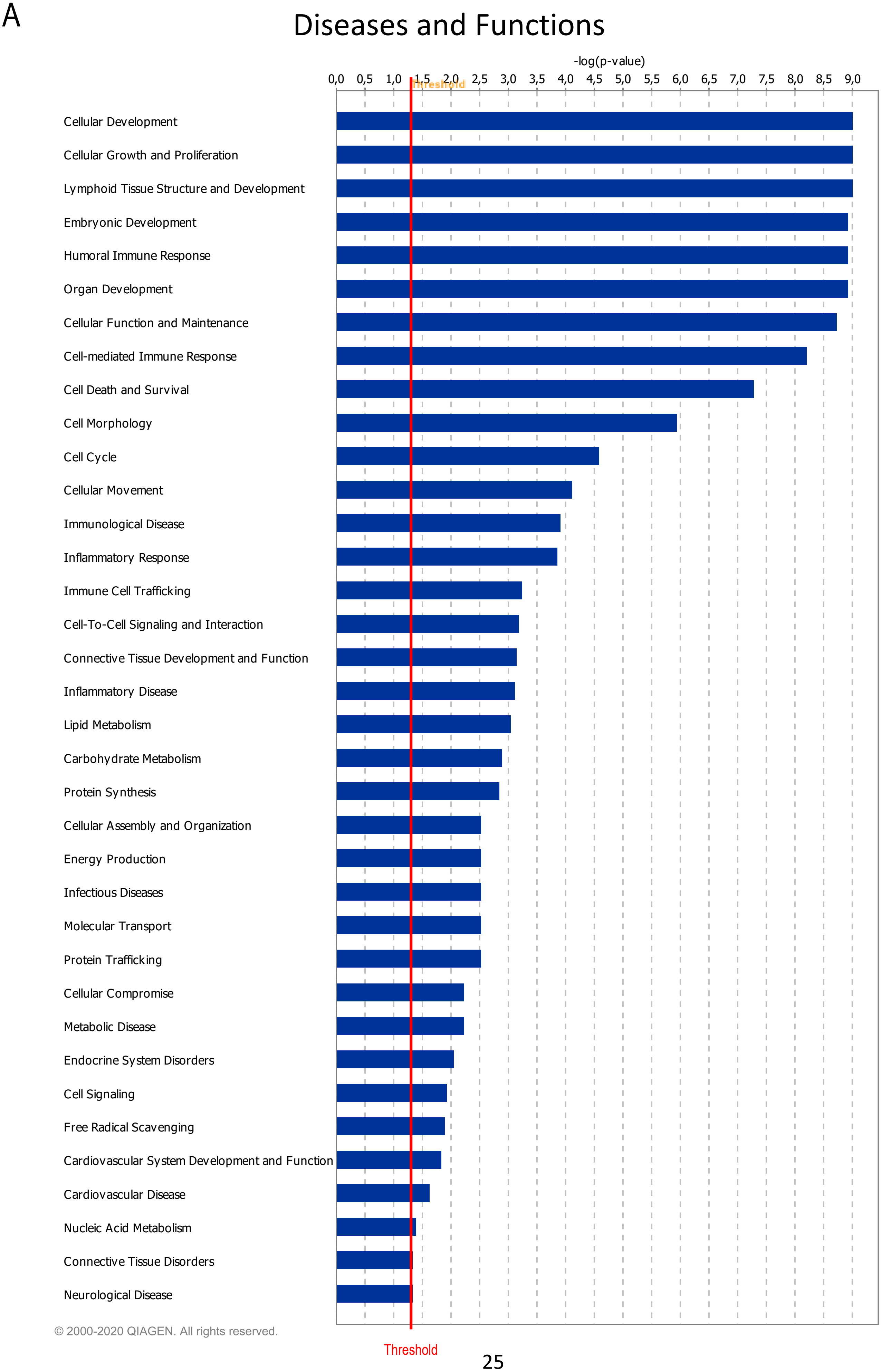

© 2000-2020 QIAGEN. All rights reserved.

Figure S4

B

Top canonical pathways

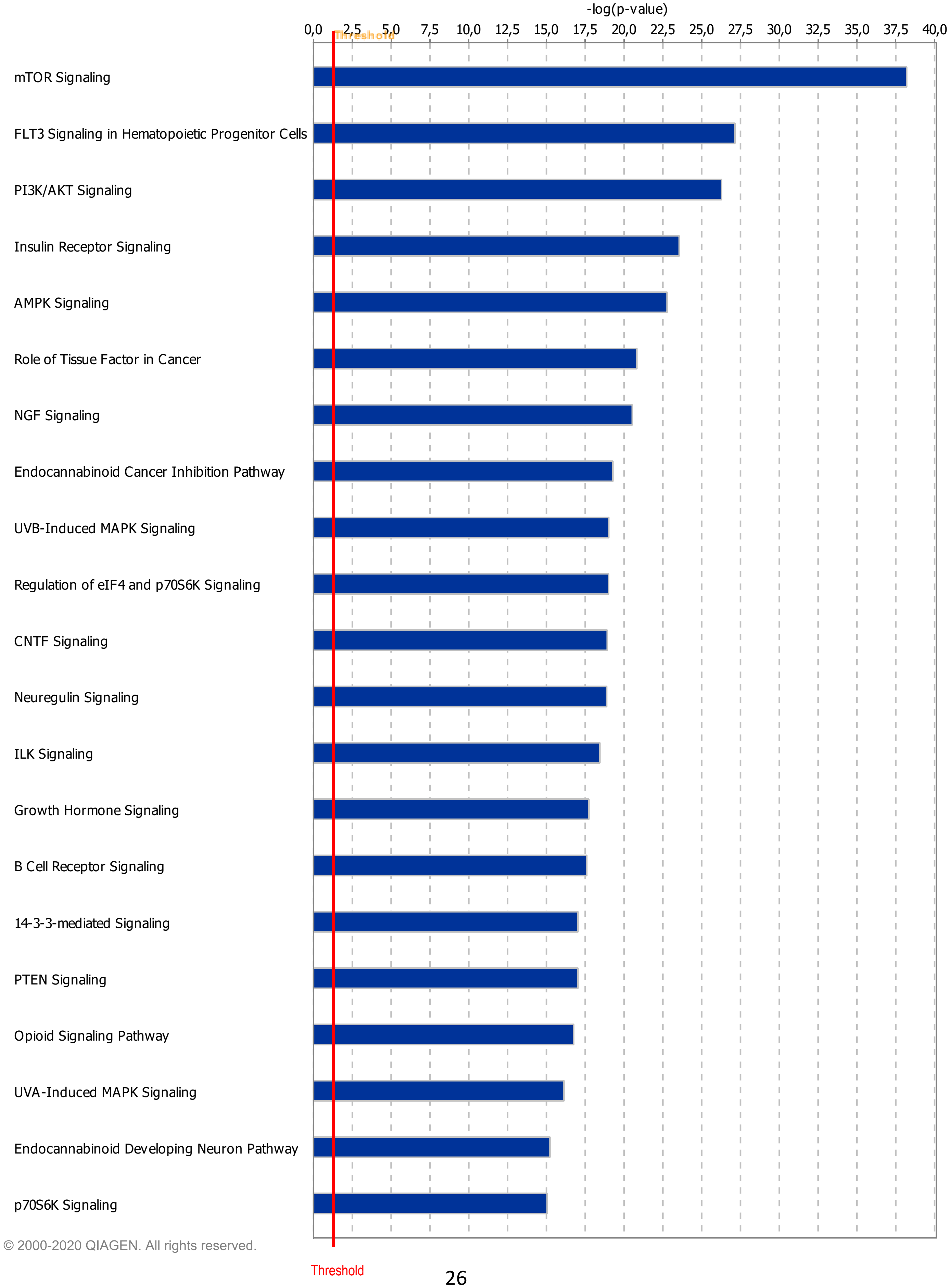

© 2000-2020 QIAGEN. All rights reserved.

Figure S4

Fig. S4: Protein Array Analysis. A) Top diseases and functions and **B)** canonical pathways from protein profiling of murine peritoneal MΦ from Sema7A$^{-/-}$ and Sema7A$^{+/+}$ mice were used following 0 h and 4 h of ZyA-induced peritonitis. Peritoneal lavages from mice were collected 0 h and 4 h after ZyA treatment and protein expression and phosphorylation were measured in peritoneal MΦ by using a protein microarray. Ratio calculated by the number of measured proteins compared with the total number of proteins involved in the pathway. Peritoneal MΦ from 4 mice/condition were pooled for analysis. The log p value was calculated by the Fisher's exact test right tailed.

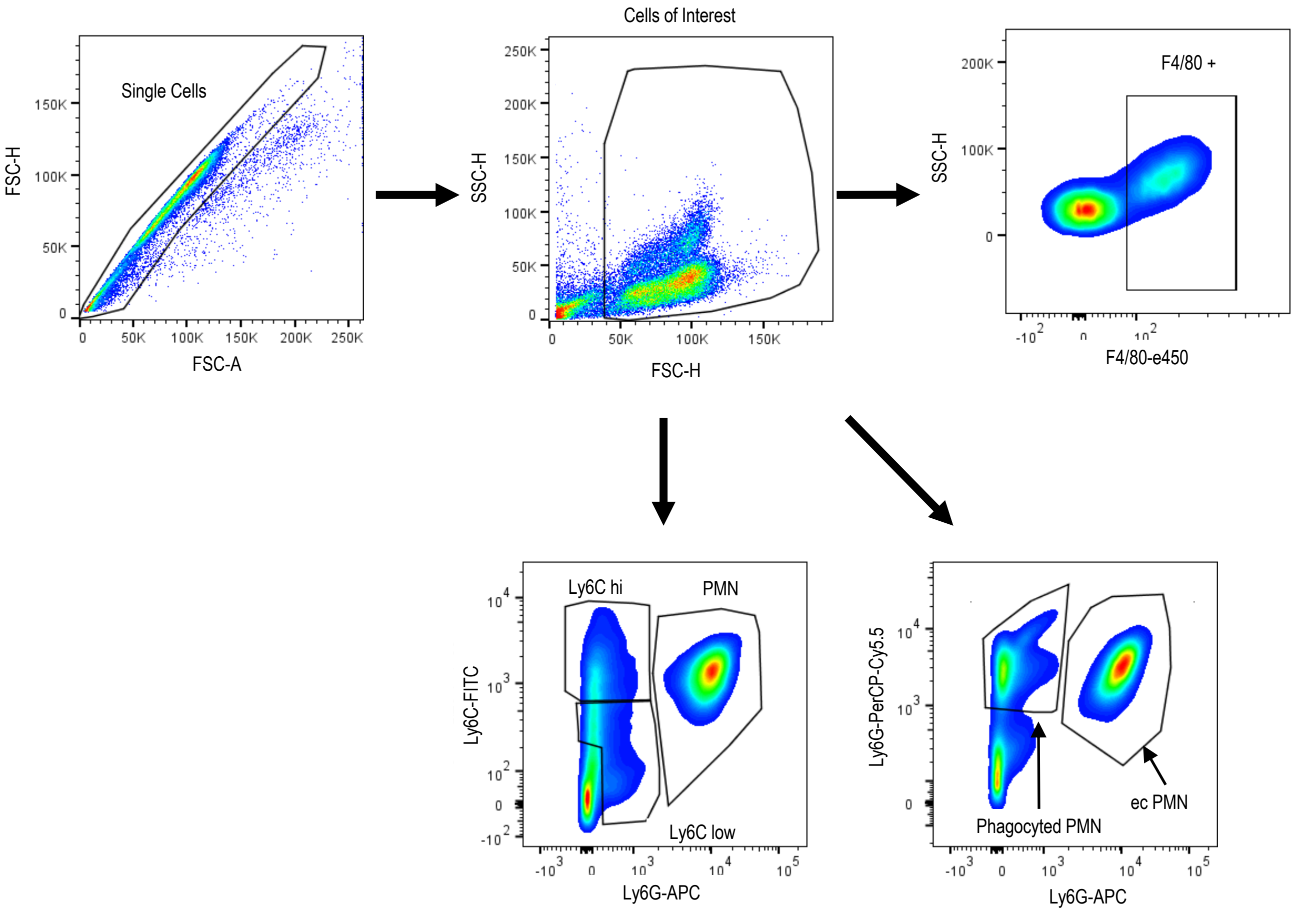
Single Cells
FSC-H
FSC-A
Cells of Interest
SSC-H
FSC-H
F4/80 +
SSC-H
F4/80-e450
Ly6C hi
PMN
Ly6C low
Ly6C-FITC
Ly6G-APC
Ly6G-PerCP-Cy5.5
Phagocyted PMN
ec PMN
Ly6G-APC

Figure S5

Fig. S5: FACS gating strategy for leukocyte differentiation. Leukocytes were gated on FSC/SSC. Leukocyte subtypes were further classified into Ly6G^{hi}, Ly6C^{hi} and Ly6C^{lo}. MΦ were F4/80 positive (+). For defining efferocytosis, the differentiation of intra- and extracellular PMN was assessed by using Ly6G-PerCP-Cy5.5 and Ly6G-APC antibodies. Phagocytized PMNs were Ly6G-PerCP-Cy5.5 positive (+) and Ly6G-APC negative (-).

		4h				
Common name	Lipidmaps ID	SEMA7A$^{+/+}$ + ZyA		SEMA7A$^{-/-}$ + ZyA		
		Mean	SEM	Mean	SEM	P value
LA	LMFA01030120	3472	300.0	2603	215.5	0.0297
Arachidonic Acid Bioactive Metabolome						
AA	LMFA01030001	1209	89.41	937.5	91.29	0.0421
TXB2	LMFA03030002	5.925	0.6395	3.999	0.462	0.0268
PGD_2	LMFA03010004	2.353	0.1377	1.824	0.1815	0.0263
PGE_2	LMFA03010003	4.488	0.4202	2.914	0.3403	0.0084
5-HETE	LMFA03060002	11.56	1.691	5.294	0.8321	0.0036
LTB_4	LMFA03020001	16.2	3.216	6.502	1.71	0.0167
6-trans-LTB_4	LMFA03020013	4.584	0.8512	2.691	0.7344	0.1084
6t.12epi-LTB_4	LMFA03020014	2.933	0.5443	2.081	0.5348	0.2767
8-HETE	LMFA03060006	0.07547	0.006864	0.06444	0.01193	0.4106
11-HETE	LMFA03060003	4.427	0.3172	3.443	0.4058	0.0617
12-HETE	LMFA03060007	1.626	0.1977	1.463	0.1716	0.5451
15-HETE	LMFA03060001	2.925	0.1745	2.371	0.1921	0.0410
LXA_4	LMFA03040001	0.08767	0.005464	0.06243	0.007965	0.0135
Eicosapentaenoic Acid Bioactive Metabolome						
EPA	LMFA01030759	103.5	8.232	64.42	7.166	0.0018
15-HEPE	LMFA03070009	0.5105	0.1008	0.4127	0.05569	0.4610
18-HEPE	LMFA03070038	0.1985	0.01107	0.1410	0.01673	0.0061
14,15-diHETE	LMFA03060077	2.097	0.2147	1.198	0.1586	0.0030
Docosahexaenoic Acid Bioactive Metabolome						
DHA	LMFA01030185	1835	134.9	1245	114.1	0.0026
PDX	LMFA04000047	0.1766	0.01382	0.1267	0.01584	0.0312
MaR1	LMFA04050001	0.08733	0.01239	0.08486	0.008163	0.8720
19,20-diHDPA	LMFA04000043	8.452	0.6911	5.444	0.9301	0.0125
7-HDHA	LMFA04000025	0.3485	0.06486	0.1192	0.02497	0.0042
17-HDHA	LMFA04000072	4.799	1.114	4.554	0.6352	0.8568

Table S3

Table S3: Lipid mediator profile. Lipid mediator levels in murine peritoneal fluids following administration of ZyA in SEMA7A$^{+/+}$ and SEMA7A$^{-/-}$ mice. Lavages were collected after 4 h and LC-MS/MS-based profiling was performed. Levels of bioactive lipid mediators and precursors derived from the AA, DHA and EPA pathways. All results are reported as ng/ml. Results represent three independent experiments with n=20 mice/group and are expressed as the mean±SEM, unpaired student's t-test.

Common name	Lipidmaps ID	4h					12h				
		WT + Vehicle		WT + Sema7A^{SL4cd}			WT + Vehicle		WT + Sema7A^{SL4cd}		
		Mean	SEM	Mean	SEM	P value	Mean	SEM	Mean	SEM	P value
LA	LMFA01030120	2539	315.0	1637	149.6	0.0325	1196	142.0	1141	113.7	0.7651
AA	LMFA01030001	1093	119.9	1543	106.0	0.0176	723.7	94.44	851.1	62.11	0.2664
TXB2	LMFA03030002	2.957	0.3047	4.420	0.5366	0.0186	5.595	1.171	2.742	0.4660	0.0353
PGD_2	LMFA03010004	1.998	0.2118	2.670	0.1941	0.0375	1.940	0.4103	1.036	0.1426	0.0467
PGE_2	LMFA03010003	2.595	0.2297	3.578	0.3891	0.0289	5.091	1.012	2.408	0.3401	0.0272
5-HETE	LMFA03060002	5.502	0.9785	7.602	1.888	0.3140	1.397	0.2423	2.163	0.2342	0.0414
LTB_4	LMFA03020001	5.066	0.8559	8.879	1.645	0.0424	2.630	2.362	0.3415	0.02429	0.3198
6-trans-LTB_4	LMFA03020013	2.524	0.3705	2.450	0.4735	0.9025	----	----	----	----	----
6t.12epi-LTB_4	LMFA03020014	4.891	0.8924	3.546	0.5816	0.2652	----	----	----	----	----
8-HETE	LMFA03060006	0.3541	0.07593	0.3061	0.06259	0.6527	0.08739	0.01993	0.06492	0.01647	0.3976
11-HETE	LMFA03060003	1.066	0.1162	0.9106	0.1194	0.3694	0.9556	0.2098	0.7268	0.1221	0.3471
12-HETE	LMFA03060007	7.502	0.9898	11.77	1.970	0.0405	1.839	0.4728	1.346	0.3605	0.4133
15-HETE	LMFA03060001	3.074	0.3932	4.754	0.7861	0.0446	1.792	0.4705	1.590	0.3169	0.7213
LXA_4	LMFA03040001	0.03857	0.005298	0.05770	0.007049	0.0370	0.01722	0.002886	0.02681	0.001631	0.0105
	Eicosapentaenoic Acid Bioactive Metabolome										
EPA	LMFA01030759	73.85	7.853	111.8	16.60	0.0310	38.78	6.398	42.60	4.513	0.6263
15-HEPE	LMFA03070009	2.374	0.3784	4.510	1.006	0.0250	0.8694	0.3032	0.9016	0.2584	0.9362
18-HEPE	LMFA03070038	0.08229	0.007420	0.1137	0.01348	0.0393	0.04147	0.005982	0.03696	0.005479	0.5891
14,15-diHETE	LMFA03060077	1.294	0.1411	2.083	0.3672	0.0312	0.3793	0.05977	0.5093	0.05944	0.1516
	Docosahexaenoic Acid Bioactive Metabolome										
DHA	LMFA01030185	917.7	78.49	1238	122.1	0.0315	450.4	70.97	471.6	41.49	0.7945
PDX	LMFA04000047	0.7235	0.1501	1.692	0.4670	0.0245	0.3377	0.1055	0.4587	0.1185	0.4686
MaR1	LMFA04050001	0.06107	0.007410	0.1058	0.02266	0.0329	----	----	----	----	----
19,20-diHDPA	LMFA04000043	8.646	1.101	13.33	2.016	0.0358	3.393	0.5008	4.564	0.4785	0.1200
7-HDHA	LMFA04000025	0.2767	0.04358	0.5531	0.1473	0.0495	0.2901	0.1948	0.1114	0.01587	0.4096
17-HDHA	LMFA04000072	9.776	1.373	12.82	2.846	0.3006	5.803	1.603	6.812	1.593	0.6630

Table S4

Table S4: Lipid mediator profile. Lipid mediator levels in murine peritoneal fluids following administration of ZyA + vehicle or Sema7A^{SL4cd} in WT mice. Lavages were collected at 4 and 12 h post ZyA injections and LC-MS/MS-based profiling was performed. All results are reported as ng/ml. Results represent three independent experiments with n=20 mice/group and are expressed as the mean±SEM, unpaired student's t-test.

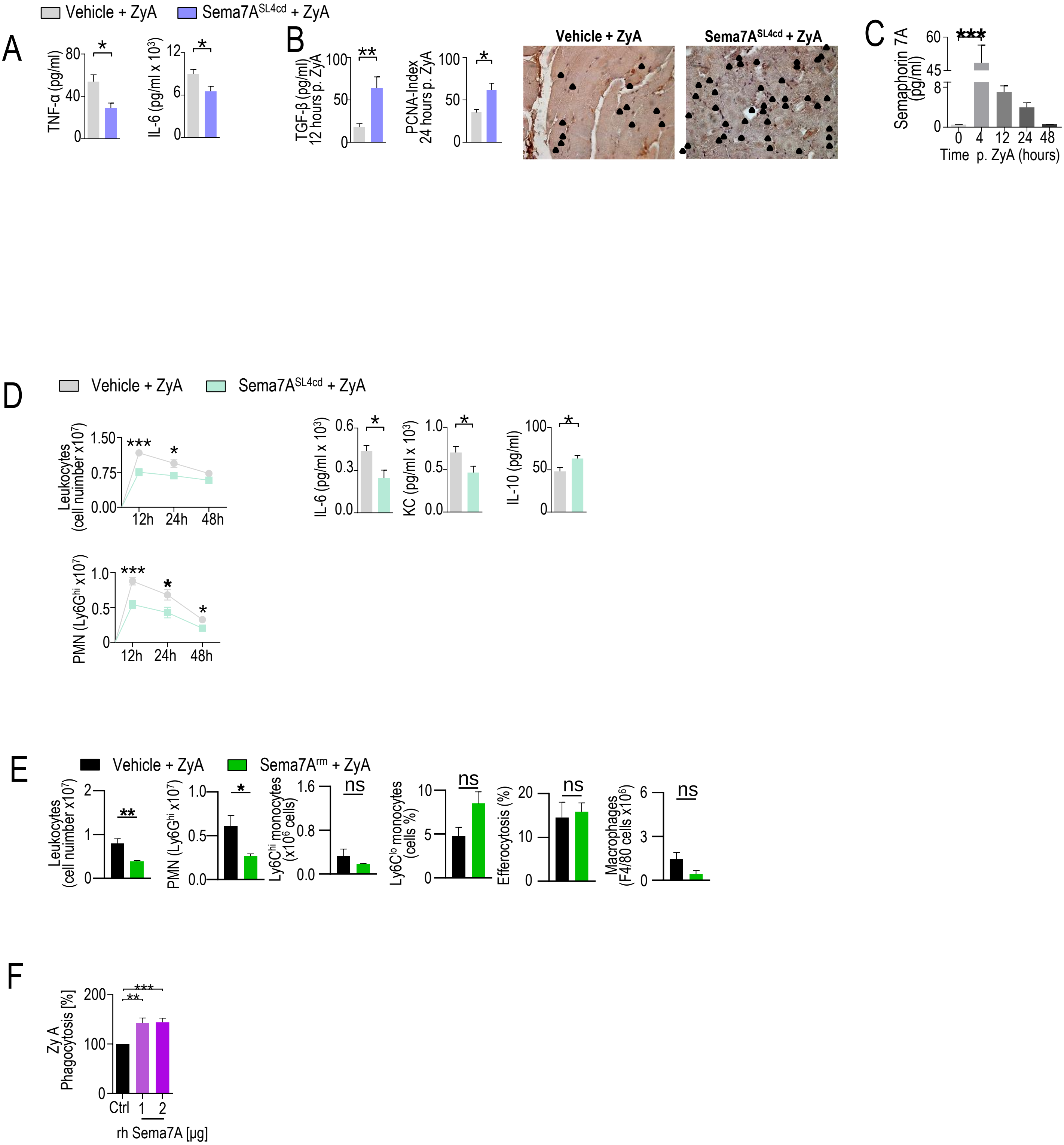

Vehicle + ZyA
Sema7A^SL4cd + ZyA
A
TNF-α (pg/ml)
IL-6 (pg/ml x 10³)
B
TGF-β (pg/ml) 12 hours p. ZyA
PCNA-Index 24 hours p. ZyA
Vehicle + ZyA
Sema7A^SL4cd + ZyA
C
Semaphorin 7A (pg/ml)
Time p. ZyA (hours)
D
Vehicle + ZyA
Sema7A^SL4cd + ZyA
Leukocytes (cell nuimber x10⁷)
12h 24h 48h
IL-6 (pg/ml x 10³)
KC (pg/ml x 10³)
IL-10 (pg/ml)
PMN (Ly6G^hi x10⁷)
E
Vehicle + ZyA
Sema7A^rm + ZyA
Leukocytes (cell nuimber x10⁷)
PMN (Ly6G^hi x10⁷)
Ly6C^hi monocytes (x10⁶ cells)
Ly6C^lo monocytes (cells %)
Efferocytosis (%)
Macrophages (F4/80 cells x10⁶)
ns
F
Zy A Phagocytosis [%]
Ctrl 1 2
rh Sema7A [µg]

Figure S6

Fig. S6: Exogenous Sema7A activates resolution programs. WT animals were injected with ZyA and treated subsequently (A-C) or after 4 h at the peak of inflammation (D) with vehicle or Sema7A^{SL4cd}. **A)** Cytokine levels of TNF-α and IL-6 in peritoneal fluids following treatment with ZyA for 4 h were measured by ELISA (n=9-17). **B)** Analysis of tissue regeneration: TGF-β levels within peritoneal lavages (n=10), PCNA response within the peritoneum (n=3). **C)** The temporal regulation of Sema7A in ZyA induced peritonitis exudates was assessed by ELISA (n=5-9). **D)** Lavages were collected at 12, 24 and 48 h. Total leukocytes were enumerated by light microscopy, PMNs were quantified by flow cytometry (n=8-13). Cytokine levels of IL-6, KC and IL-10 were measured in peritoneal fluids by ELISA. **E)** In a different set of experiments, WT animals were injected with ZyA and treated subsequently with Sema7A recombinant mouse protein (R&D systems) and lavages were collected at 4h. **F)** The rate of MΦ clearance of fluorescence-labeled ZyA particles after stimulation with the recombinant Sema7A protein was assessed photometrically (n=8). The results represent three independent experiments and are expressed as the mean±SEM, unpaired two-tailed Student'st-test, *P<0.05; **P<0.01; ***P<0.001.

Dataset S1: Protein and phosphorylation profile of murine peritoneal macrophages. (see separate Excel file)
